# Poplar trees reconfigure the transcriptome and metabolome in response to drought in a genotype- and time-of-day-dependent manner

**DOI:** 10.1186/s12864-015-1535-z

**Published:** 2015-04-21

**Authors:** Erin T Hamanishi, Genoa LH Barchet, Rebecca Dauwe, Shawn D Mansfield, Malcolm M Campbell

**Affiliations:** Faculty of Forestry, University of Toronto, 33 Willcocks St., Toronto, ON M5S 3B2 Canada; Centre for the Analysis of Genome Evolution and Function, University of Toronto, 25 Willcocks St., Toronto, ON M5S 3B2 Canada; Department of Wood Science, University of British Columbia, 4030-2424 Main Mall, Vancouver, BC V6T 1Z4 Canada; Department of Cell & Systems Biology, University of Toronto, 25 Willcocks St., Toronto, ON M5S 3B2 Canada; Department of Biological Sciences, University of Toronto Scarborough, Toronto, ON M1C 1A4 Canada; Current address: Département des sciences agronomiques et écologiques, Université de Picardie Jules Verne, Amiens, 80039 cedex France

**Keywords:** Balsam poplar, Drought, Metabolome, Transcriptome, Trees, Forests

## Abstract

**Background:**

Drought has a major impact on tree growth and survival. Understanding tree responses to this stress can have important application in both conservation of forest health, and in production forestry. Trees of the genus *Populus* provide an excellent opportunity to explore the mechanistic underpinnings of forest tree drought responses, given the growing molecular resources that are available for this taxon. Here, foliar tissue of six water-deficit stressed *P. balsamifera* genotypes was analysed for variation in the metabolome in response to drought and time of day by using an untargeted metabolite profiling technique, gas chromatography/mass-spectrometry (GC/MS).

**Results:**

Significant variation in the metabolome was observed in response the imposition of water-deficit stress. Notably, organic acid intermediates such as succinic and malic acid had lower concentrations in leaves exposed to drought, whereas galactinol and raffinose were found in increased concentrations. A number of metabolites with significant difference in accumulation under water-deficit conditions exhibited intraspecific variation in metabolite accumulation. Large magnitude fold-change accumulation was observed in three of the six genotypes. In order to understand the interaction between the transcriptome and metabolome, an integrated analysis of the drought-responsive transcriptome and the metabolome was performed. One *P. balsamifera* genotype, AP-1006, demonstrated a lack of congruence between the magnitude of the drought transcriptome response and the magnitude of the metabolome response. More specifically, metabolite profiles in AP-1006 demonstrated the smallest changes in response to water-deficit conditions.

**Conclusions:**

Pathway analysis of the transcriptome and metabolome revealed specific genotypic responses with respect to primary sugar accumulation, citric acid metabolism, and raffinose family oligosaccharide biosynthesis. The intraspecific variation in the molecular strategies that underpin the responses to drought among genotypes may have an important role in the maintenance of forest health and productivity.

**Electronic supplementary material:**

The online version of this article (doi:10.1186/s12864-015-1535-z) contains supplementary material, which is available to authorized users.

## Background

Drought is one of the most significant environmental stresses that can impinge on the growth and productivity of forests. Recently, more severe and frequent drought events have been associated with increased global forest-dieback [1]. In North America, severe drought and higher summer temperatures have been linked with the widespread die-off of *Populus tremuloides* [2].

Trees of the genus *Populus* are often characterized by their high productivity [3,4]. The rapid growth rates attributed to poplars are often associated with significant water requirements. Thus, the growth, productivity and survival of poplars is often dependent on water availability [5-7].

In response to water limitation, plants may exhibit adaptation at morphological, physiological and biochemical level to contend with the abiotic stress. For instance, at the physiological level, strategies to contend with reduced water availability can vary from isohydric to ansiohydric [8]. Poplar trees generally respond by closing their stomata during periods of water limitation to reduce water loss, thus limiting the photosynthetic capacity of the trees [9]; however, this response to water limitation is often variable among poplar trees.

Among three closely related poplar genotypes, Larcheveque et al. [10] found that the response to drought varied at the physiological level. Specifically, two hybrid *Populus balsamifera* and one native *P. balsamifera* genotypes had variable growth rates and water use efficiencies under drought conditions [10].

Previous studies have identified significant variation at the molecular level among poplar trees that may underpin variation at the morphological and physiological level. Large-scale microarray experiments studying water-deficit stress have identified many transcripts with known roles in stress tolerance in *Populus* [11-15]. For example, among six genotypes of *P. balsamifera*, Hamanishi et al. [14] observed significant variation in patterns of transcript accumulation. The variation in the drought transcriptomes among the six *P. balsamifera* genotypes was correlated with their ability to maintain growth following water limitation [14], highlighting the complexity in the drought response among poplars.

The great intra- and interspecific variation seen among poplar species is also reflected at the biochemical level. In trees, metabolites involved in osmotic adjustment, protection and stabilization of cellular structure and redox regulation are often involved in drought responses [16]. For example, the amino acids proline (Pro), valine (Val) and isoleucine (Ile), carbohydrates such as sucrose, raffinose family oligosaccharides (RFO) and sorbitol, polyols, and organic acids have been shown to vary in abundance in response to drought [11]. Elevated levels of sucrose were observed in leaf tissue of water-stressed *Populus tomentosa* [17]; whereas a combination of glucose, fructose, and sucrose accumulated in *Populus* hybrids in response to drought [18]. Some of these compounds are thought to function as osmolytes, maintaining cell turgor and stabilisation of cellular proteins [19]. Similarly, raffinose and the RFO accumulate in response to water-stress, and are hypothesised to be osmoprotectants, with the capacity for membrane and enzyme stability [11,20], along with a putative role as hydroxyl radical scavengers.

Proline accumulation has long been associated with stress tolerance in plants, and is likely one of the most widely distributed osmolytes among plants and animals [17,21]. Similar to carbohydrates, proline is hypothesised to aid in the osmotic adjustment in response to drought; however, proline is also hypothesised to have roles in reactive oxygen species (ROS) scavenging and membrane stability. Proline has been shown to accumulate in severely water-stressed mature *Populus nigra* leaves [18,22]; whereas no significant increase in proline accumulation was observed in field-grown, drought-treated *Populus* hybrids [6].

Organic acids have also been implicated in the biochemical response to drought. For example, malic acid increased in abundance under mild periods of water stress [6,19,23]. Unlike carbohydrate and amino acid accumulation, malic acid accumulation may be a function of the stomatal system in plants rather than being osmotically active [24].

As the response to drought stress is not simply the product of the drought-responsive transcriptome, complexity in the whole-plant response to drought is the result of the interactions between genes, transcripts, proteins, metabolites, and the environment. The model plant genus *Populus* provides an opportunity to explore the relationship between the drought transcriptome and the drought metabolome. In keeping with this, the relationship between the transcriptome and metabolome for specific metabolic pathways in *Populus* has also been characterised in response to salt stress, revealing the importance of control mechanisms for osmotic adjustment [19,25].

In order to test hypotheses related to intra-specific variation in drought responses in *Populus*, the transcriptomes and metabolomes of six genotypes of *P. balsamifera* were examined. Shared *versus* genotype-specific *P. balsamifera* drought transcriptomes were identified [14] and superimposed onto metabolome variation. This approach identified important pathways in the drought response, and highlighted genotypic-specific responses that provide insight into different mechanisms of acclimation to water-limiting conditions.

## Methods

### Plant material and experimental design

*Populus balsamifera* ramets were grown in a climate controlled growth chamber at the University of Toronto using conditions as described by Hamanishi et al. [14]. Un-rooted cuttings of six *P. balsamifera* genotypes (AP-947, AP-1005, AP-1006, AP-2278, AP-2298 and AP-2300; Alberta Pacific, Boyle, Alberta) were propagated and grown under well watered conditions for 9 weeks, at which point, water-deficit stress was imposed on half the trees by withholding water, while temperature, light, and relative humidity were held constant.

Foliar tissue was harvested for metabolite and transcriptome analysis 15 days after the onset of the water withdrawal. For the transcriptome analysis, the first fully expanded [leaf plastochron index (LPI = 7)], mature leaf was collected from each tree; three leaves were pooled to create a single replicate. Triplicate replicates were collected for each genotype and treatment combination at pre-dawn (PD; 1 hour before the light period) and mid-day (MD; middle of the light period). Leaves were immediately flash frozen in liquid nitrogen, and then ground to a fine powder in preparation for RNA isolation, as described by Hamanishi et al. [24]. For the metabolite analysis, a single mature, fully expanded leaf was collected from each tree (n = 10 per genotype per treatment at MD and PD) and immediately flash frozen. Harvested foliar tissue was weighed to determine fresh weight (FW), subsequently freeze-dried, and weighed again to determine dry weight (DW).

### Non-targeted metabolic profiling by gas chromatography/mass spectrometry

Metabolite extraction was performed using a methanol/chloroform-based extraction protocol as described by Robinson et al. [19,25]. Four to 10 biological replicates were sampled per genotype, treatment, time of day (Additional file 1: Table S1). Approximately 0.5 mL of sample was extracted in 1300 μL 97% methanol with the internal standard ortho-anisic acid (0.62 mg mL^−1^) for 15 minutes at 70°C prior to centrifugation at 17,000 g for 10 minutes. The supernatant was transferred to a new 1.5-mL tube. 130 μL chloroform and 270 μL distilled, deionized water was added and the tube was gently shaken prior to centrifugation at 17,000 g for 5 minutes. A 400 μL aliquot of the upper polar phase was transferred to a new 1.5 mL tube and dried overnight at 30°C in a Vacufuge (Eppendorf).

Samples were then derivatised for gas chromatography/mass spectrometry (GC/MS) analysis by resuspension in 50 μL methoxyamine hydrochloride solution (20 mg mL^−1^ in pyridine) and incubated at 37°C for 2 hours. 10 μL of n-alkane standard and 70 μL of N-methyl-N-trimethylsilytriflouroacetamide (MSTFA) was added, and incubated at 37°C for 30 minutes with constant agitation. Samples were then filtered through filter paper and allowed to rest at room temperature until GC/MS analysis.

GC/MS analysis was conducted on a ThermoFinnigan Trace GC-PolarisQ ion trap MS, fitted with an AS2000 auto-sampler and a split-injector (Thermo Electron Co., Waltham, MA, USA). The GC was equipped with a Restek Rtx-5MS column (fused silica, 30 m, 0.25 mm ID, stationary phase: 5% diphenyl, 95% dimethyl polysiloxane). The GC conditions were set with an inlet temperature of 250°C, helium carrier gas at a constant flow rate of 1 mL min^−1^, injector split ratio 10:1, resting oven temperature at 70°C and a GC/MS transfer line temperature of 300°C. After a sample injection of 1 μL, the oven temperature was held at 70°C for 2 minutes prior to ramping to 325°C at a rate of 8°C min^−1^. The temperature was held at 325°C for 6 minutes before cooling to the initial resting oven temperature, prior to the next run.

For MS analysis in the positive electron ionisation mode an ionization potential of 70 eV was used and the foreline was evacuated to 40 mTorr with helium gas flow in to the chamber set at a rate of 0.3 mL min^−1^ and the source temperature was held at 230°C. Detector signal was recorded from 3.35-35.5 minutes after the injection, and, with a total scan time of 0.58 s, ions were scanned across the range of 50–650 mass units.

### Metabolome: data processing and statistics

The raw metabolite data generated by GC/MS for each metabolite was normalised through comparison to internal standards and normalised to freeze-dried DW for each tissue sample. Raw-data was processed using XCMS as described by Krasensky and Jonak [14]. Descriptive statistics were calculated using R 2.14.1 [26]. For subsequent analyses, the metabolite data were log_10_ transformed. The dataset comprised 87 metabolites, 181 samples (n = 4-10 per genotype per treatment per time of day).

Metabolic profiles for all samples were subjected to hierarchical cluster analysis (HCA) using Pearson correlation coefficient [27,28] to search for metabolic similarities and differences among samples and metabolites. The uncertainty associated with HCA was assessed generating a consensus dendrogram on 1,000 bootstrap replicates using the R package pvclust [29]. Over-representation of a given metabolite class within a cluster was determined using Fisher’s exact test in R [26]. Statistical significance was calculated using a three-way analysis of variance (ANOVA). The *p* values were corrected for multiple hypothesis testing using the false discovery rate (FDR) procedure of Benjamini and Hochberg [30]. A *p* value of < 0.05 was considered statistically significant.

### RNA isolation and analysis

RNA isolation and microarray analysis was performed as described by Hamanishi et al. [14]; all samples were uploaded to Gene Expression Omnibus (http://www.ncbi.nlm.nih.gov/geo/); accession number GSE21171. For the purposes of subsequent analyses, the global drought transcriptome was considered to include all transcripts significant for a treatment-main effect (*p* < 0.05) with no log_2_ (fold-change) cutoff. Weighted co-expression network analysis (WGCNA) was performed using the R statistical package WGCNA with a power of 7 [31]. Functional annotations were assigned based on the most recent version probe-set annotations from Affymetrix (NetAffx build 32). Networks generated with WGCNA were plotted using Cytoscape [32]. Analysis of gene ontology (GO) term enrichment was calculated by comparing the number of annotations within the list of query transcripts to all annotated transcripts on the Poplar Affymetrix Genome Array. Statistical significance was calculated using Fisher’s exact test in R [26], and applying the Benjamini-Hochberg correction to adjust for FDR. Overrepresentation of GO Slim terms was confirmed and plotted using AgriGO [33]. Molecular pathways relevant to the drought transcriptome/metabolome were previously characterised in Kyoto Encyclopedia of Genes and Genomes (KEGG; [34-36]).

## Results and discussion

### Populus balsamifera genotypes were subjected to water withdrawal to induce a drought response

To investigate the impact of drought-like conditions on the abundance of *Populus balsamifera* metabolites, six genotypes (AP-947, AP-1005, AP-1006, AP-2278, AP-2298 and AP-2300) were exposed to a prolonged period of water withdrawal. All plants were grown under the same controlled growth conditions for 9 weeks, after which half of the plants continued to receive water (well watered) and the other half received no water (water deficit). This divergence in treatment continued for 15 days, at which point foliar tissue for metabolic and transcriptome analysis was collected at PD and MD, and aboveground biomass and relative water content (RWC) was recorded.

**Figure 1:**
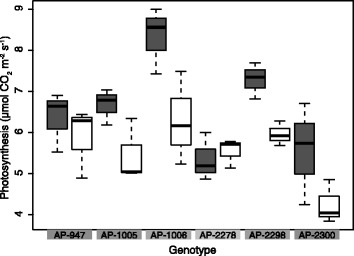
Box-plot representing net photosynthetic rate (μmol CO_2_ m^−2^ s^−1^) for genotype AP-947, AP-1005, AP-1006, AP-2278, AP-2298 and AP-2300. Well watered samples (filled); water-deficit-treated samples (empty) (n = 3 per treatment per genotype). The midline of the box represents the median value for photosynthesis, the upper and lower bounds of the box represent the interquartile range, and the whiskers extend to the most extreme values that are not outliers.

Under conditions of water deficit, significant declines in aboveground biomass (Table 1) and RWC were observed in most genotypes. In well watered conditions, AP-2278 had significantly lower aboveground biomass, and AP-2300 had the highest aboveground biomass relative to all other genotypes (Table 1). Stomatal conductance significantly decreased in all genotypes, with the greatest decline observed in AP-1006, whereas genotype AP-2278 had the smallest reduction in stomatal conductance in response to the imposition of water-deficit condition. No correlation between aboveground biomass and decline in stomatal conductance was observed among the six genotypes. More specifically, larger plants (e.g., AP-2300) did not show a greater reduction in stomatal conductance under water-deficit conditions. Net photosynthetic rate decreased in response to water-deficit conditions after 15 days of water withdrawal (treatment main effect; ANOVA, *p* < 0.05); however, a significant decline only occurred in genotypes AP-1005, AP-1006, and AP-2298 (Welch’s two-sample *t*-test, *p* < 0.05; Figure 1). Reduced photosynthetic rates observed in the chamber-grown seedlings are likely attributable to the lower light intensity in the growth chamber as compared to ambient levels in field-grown seedlings or trees.

**Table 1:** **Aboveground biomass for well watered and water-deficit treated Populus balsamifera genotypes (n = 6–12 for each genotype, treatment)**

**Genotype**	**Aboveground dry biomass, well watered (g)**	**Aboveground dry biomass water deficit (g)**	**t-stat**	**p-value**	
AP-947	3.12	2.17	3.04	0.008	*
AP-1005	4.23	2.89	2.62	0.007	*
AP-1006	3.01	2.18	2.24	0.042	*
AP-2278	2.12	1.85	0.74	0.472	
AP-2298	2.46	2.26	0.40	0.699	
AP-2300	5.27	3.62	3.52	0.004	*

*p value < 0.05.

### Variation in populus balsamifera metabolite profiles was evident

To differentiate between genotypic (G), treatment (T), and time-of-day (D) effects, metabolic profiles of *P. balsamifera* were analysed using gas chromatography/mass spectrometry (GC/MS). Trend analysis was restricted to 87 metabolites that were identified across all samples (n = 4-10 per genotype per treatment per time of day), which represented both known and unknown metabolites (Additional file 1: Table S1; Table S2). A large degree of variation in metabolite abundance profiles among samples was observed, as indicated by the dendrogram. Notably, both genotype and treatment appeared to play an important role in the segregation of samples (Additional file 2: Figure S1). The metabolite profiles from water-deficit samples of AP-1005 and AP-2278 appeared most different from the other metabolomes. Specifically, the metabolite profile for AP-1005 was separated by treatment rather than time of day. Additionally, samples of AP-947 and AP-2300 clustered in a genotype-wise fashion, regardless of time of day or treatment.

Although the metabolomes were highly variable among samples, further investigation of the relationship among metabolites revealed 13 significant clusters of metabolites that had a high degree of similarity in their abundance profiles across all samples (Additional file 3: Figure S2), as determined by HCA. Unique clustering of these metabolites may be indicative of a different mechanism that governs their regulation. For example, three of the 13 clusters had significant over-representation of a given metabolite class (Fisher’s exact test; *p*_*adj*_ < 0.05). Specifically, cluster II was predominantly carbohydrates (*p*_*adj*_ = 0.00366), cluster IX was all organic acids (*p*_*adj*_ = 0.00245), and cluster XII was primarily amino acids (*p*_*adj*_ = 0.000251).

A three-way factorial (ANOVA) identified metabolites that had significantly different abundance in response to drought treatment (T main effect), genotype (G main effect), time of day (D main effect), as well as any interaction between the three experimental factors (Table 2, Additional file 1: Table S3). Similar to the HCA results among metabolites; significant variation in the metabolic profiles was attributable to genotype. A large proportion of metabolites had differential abundance among genotypes (n = 79; *p* < 0.05; Table 3). Of the 79 metabolites with significant variation among genotypes, no interaction with any factor was found for 38 metabolites.

**Table 2:** **Number of metabolites with significant main effects or interactions (n = 87 metabolites)**

	**Number of metabolites**	**Percent (%) of metabolites**
Genotype (G)	79	91.95%
Treatment (T)	40	45.98%
Time of day (D)	11	12.64%
Genotype:treatment (GxT)	41	47.13%
Genotype:time of day (GxD)	6	5.75%
Treatment:time of day (TxD)	15	17.24%
Genotype:treatment:time of day (GxTxD)	0	0.00%

*p*_*adj*_-value cutoff = 0.05 (Benjamini-Hochberg).

**Table 3:** **Metabolites with significantly different abundance levels in response to drought (ANOVA,** ***p***_***adj***_ **-value < 0.05)**

**Group**	**Metabolite**	**Log**_**2**_ **(fold-change)**
Amino acid	Aspartic acid	−0.68
	L-Isoleucine	3.32
	L-Threonine	−0.39
	NI (Amino acid; 2)	−0.26
Carbohydrate	Fructose (2)	−0.16
	Glycerol	−0.58
	Melibiose	0.3
	NI (5C sugar; 2)	−1.18
	NI (6C sugar; 1)	−0.16
	Raffinose	1.13
	Sucrose	0.28
Organic acid	Benzoic acid	1.13
	Citric acid	1.07
	Fumaric acid	−1.3
	Glycolic acid	−0.39
	Malic acid	−0.12
	Malonic acid	−1.58
	Quinic acid	−0.37
	Shikimic acid	−0.55
	Succinic acid	−0.97
	Threonic acid	−0.25
	Threonic acid 1,4-lactone	−0.57
Phenolic	Catechol	0.45
	Quercitin	−0.24
	Salicin	0.83
	Salicyl_alcohol	1.56
Sugar alcohol	Galactinol	0.52
	Myo-inositol	0.15
Not identified	NI (2)	−0.14
	NI (3)	−0.43
	NI (4)	0.36
	NI (5)	0.69
	NI (7)	0.15
	Ni (9)	0.24
	NI (10)	1.36
	NI (11)	0.53
	NI (15)	0.97
	NI (18)	0.39
	NI (20)	0.55
	NI (25)	0.53

**Figure 2:**
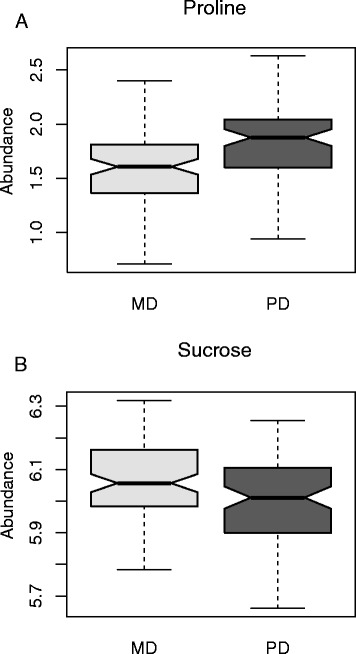
Time of Day main effect observed for **(A)** proline and **(B)** sucrose between mid-day (MD; light grey) and pre-dawn (PD; dark grey).

ANOVA analysis also revealed a small subset (n = 11) of metabolites that varied significantly in abundance in response to time of day (Figure 2). However, a larger number of metabolites (n = 15) had abundance that varied significantly in response to water-deficit treatment in a time-of-day dependent fashion (TxD interaction; Table 2; Additional file 4: Figure S3). Notably, proline had a significantly higher abundance at PD relative to MD (Figure 2A). Conversely, sucrose had higher abundance at the MD time point (Figure 2B). In plants, sucrose concentrations fluctuate diurnally, with increased abundance during light conditions [37-40].

### A populus balsamifera drought metabolome was identifiable

**Figure 3:**
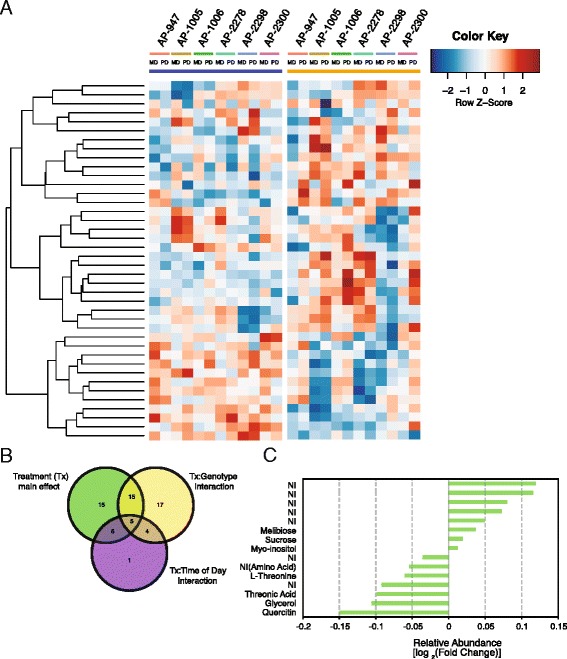
Metabolite accumulation levels for treatment main effect and treatment x genotype interaction. **(A)** Hierarchal clustering of metabolites significant for treatment main effect across all genotypes at two different time-points [pre-dawn (PD) and mid-day (MD)]. **(B)** Venn diagram demonstrating the number of metabolites that are significant for treatment main effect or a 2-way interaction. **(C)** Mean log_2_ (fold-change) of metabolite abundance for metabolites that are significant for treatment main effect only.

Water withdrawal induced significant changes in metabolite abundance. Four to 10 biological replicates were analysed per treatment, per genotype and per time of day. ANOVA analysis, taking into account intra-replicate variation (residual error, Additional file 1: Table S3), identified 40 metabolites with different abundance levels in response to drought. Twenty-one metabolites increased in abundance and 19 decreased in abundance (*p* < 0.05; Table 3; Figure 3A). No general class of metabolites responded to drought. For example, the amino acid (AA) class had variable response to drought. The contribution of amino acids in *Populus* clones is thought to be small relative to the effect of carbohydrates and other osmolytes [41]. However, isoleucine had the largest fold increase in abundance in response to drought of any metabolite assessed, and was the only branched chain amino acid (BCAA) to be analysed, whereas aspartic acid and threonine decreased in abundance in the drought-treated samples. Increased accumulation of BCAAs has been observed in other organisms including *Arabidopsis* [42] and various wheat cultivars [43]. Although increased accumulation of BCAAs has frequently been observed in response to abiotic stress, little is known about their role in stress tolerance; however, accumulated BCAAs may serve as a substrate for the synthesis of other stress-induced proteins and may act as signalling molecules in response to drought stress [44].

Two organic acids, representative of TCA cycle intermediates, succinic and malic acid, had a general decline in abundance; whereas raffinose and galactinol were some of the most highly accumulated metabolites in response to water-deficit conditions (Table 3). Although a general decline was observed in abundance of malic acid, patterns of accumulation in response to drought in *Populus* are often varied; both increased and decreased levels of accumulation in response to drought have been observed [41,45]. Malic acid is a very abundant organic acid in plants, and its role is likely not restricted to the citric acid cycle [46]. Sugars have previously been shown to increase in abundance in response to water-stress, having an important role in the osmotic adjustment [47,48]. Raffinose and galactinol have been hypothesised to be osmoprotectants in drought-stress conditions, and have frequently been implicated in the drought response in plants [17,20].

Metabolites commonly associated with drought or stress in plants constituted the core drought metabolome (i.e., T main effect). For example, substantial accumulation of raffinose and galactinol, important stress related carbohydrates, occurred in drought treated trees. Notably, of the 40 metabolites that were significant for T main effect, only 15 did not show any significant interactions (i.e., TxG or TxD; Figure 3B). Carbohydrates, a sugar alcohol, and some unknown metabolites had increased abundance in water-deficit conditions, whereas decreased abundance was exhibited by a variety of metabolites representative of different metabolite classes (Figure 3C). As indicated by the large proportion of metabolites significant for TxG or TxD interactions, the accumulation of metabolites was not simply due to the imposition water-deficit stress, rather, metabolite accumulation was a complex response shaped by genotype and time of day. The variation in metabolite accumulation across genotypes and at different time-points could be exploited to further investigate the unique responses of *P. balsamifera* genotypes.

### The drought metabolome varied among P. balsamifera genotypes

While a large proportion of metabolites had significant response to water-deficit treatment, many of these varied in a genotype- (G) or a time-of-day- (D) dependent manner (Figure 3B, Additional files 3 and 4: Figure S2 and Figure S3). The abundance of 41 metabolites was significantly impacted by TxG interaction (Table 2; Additional file 5: Figure S4). Certain metabolites had opposite patterns of accumulation in response to drought (i.e., higher abundance in one genotype and lower abundance in another genotype). Of note, glucose had elevated abundance levels in AP-947 and AP-1006, but decreased abundance levels in the remaining four genotypes in response to water-deficit conditions. Similarly, galactinol was significant for a G x T interaction (*p* = 0.0259); the highest level of galactinol accumulation was observed in drought-treated samples of genotype AP-947 and AP-2278. Other metabolites that had a significant TxG interaction demonstrated consistent directionality of response to water-deficit stress among the six genotypes. For example, glycolic and threonic acids, two metabolites belonging to cluster IX (Additional file 3: Figure S2), decreased in abundance in response to water-deficit conditions in all genotypes, with substantial reductions observed in genotype AP-1005 and AP-2278. Moreover, half of the metabolites that had significant differences in abundance between treatments (T main effect) also varied in response to genotype (n = 20; Figure 3B) confirming the importance of genotype in defining the drought response observed among samples.

Ten drought-responsive metabolites had significant differences in abundance for a TxD interaction, indicative of the variation in metabolite level observed between pre-dawn and mid day. Raffinose abundance was significant for a TxD interaction, having ~2-fold increase in accumulation in response to water-deficit at MD (*p* = 0.0122), but no significant change in abundance at PD (Additional file 4: Figure S3).

**Figure 4:**
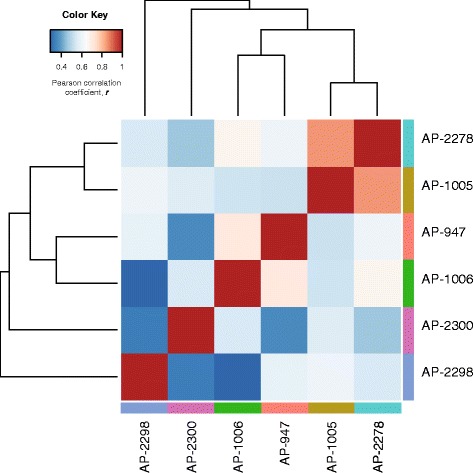
Variation in the drought metabolome among six genotypes of *P. balsamifera* represented by a Pearson correlation coefficient (PCC) heatmap. Differential abundance [log_2_ (fold-change)] for metabolites significant for treatment main effect (ANOVA, *p* < 0.05) are represented. The PCC value was calculated for each pair-wise comparison among genotypes, and is represented by the colour in the given cell. All genotypes are represented on both the x- and y-axis in the same order.

A notable feature of the *P. balsamifera* drought metabolome was the magnitude of variation observed between samples. On average, peak signal intensity (non-transformed data) varied ~3000-fold between minimum and maximum peak intensity for any given metabolite. Similarly, the magnitude of variation in metabolite accumulation between water-deficit and well watered samples varied considerably. Among the metabolites whose accumulation had a significant T main effect, the fold-change variation ranged from ~3 fold decrease in malonic acid accumulation to ~10 fold increase in isoleucine accumulation. Overall variation in the drought metabolome was examined by Pearson correlation comparison of the log_2_ (fold-change) of the water-deficit metabolome of the six *P. balsamifera* genotypes. This analysis revealed which genotypes had metabolome responses that were more equivalent to others (Figure 4; Additional file 1: Table S4). Genotypes AP-1005 and AP-2278 had the most similar drought metabolomes (r = 0.845; *p* < 0.05), whereas genotypes AP-2300 (r < 0.550) and AP-2298 (r < 0.606) were most divergent when compared to all other genotypes (Figure 4; Additional file 1: Table S4).

The magnitude of drought-induced changes in metabolite abundance among the six *P. balsamifera* genotypes had a high degree of variation (Additional file 6: Figure S5A). The largest absolute magnitude change in drought responsive metabolites occurred in AP-1005 (mean = 0.361, standard deviation = 0.340) and AP-2278 (mean = 0.327; standard deviation = 0.224), whereas the smallest magnitude change was observed in genotype AP-1006 (mean = 0.184; standard deviation = 0.223).

### There were correlations between drought-responsive metabolites and specific components of transcriptome remodelling

To assess relationships between drought-responsive metabolites and transcripts, the metabolomes and transcriptomes of *P. balsamifera* were compared. These analyses made use of previously-reported drought-responsive transcriptome data for *P. balsamifera* [14]. Quantitatively, there was a high level of congruence between the metabolome and the transcriptome, where larger magnitude changes in the transcriptome corresponded with larger magnitude changes in the metabolome, with the notable exception for genotype AP-1006 (Additional file 6: Figure S5). Specifically, genotype AP-1006 and AP-2278 had significantly larger magnitude change in the drought transcriptome relative to all other genotypes (Bonferroni’s *p* < 0.001; Additional file 6: Figure S5B); whereas, the absolute magnitude change observed in the metabolome for AP-1006 and AP-2278 was among the smallest and largest, respectively. This suggests that coordination of the transcriptome and metabolome is variable among genotypes, and that the overall magnitude change in metabolite abundance does not necessarily reflect the magnitude of transcriptome variation resulting from water-deficit treatment.

A correlation matrix of all pair-wise comparisons among drought responsive metabolites and transcripts revealed 747 transcripts that were significantly correlated with at least one metabolite (Pearson correlation coefficient, |r| > 0.60, *p* < 0.05), based on the similarity of abundance profiles across all samples (Additional file 7: Figure S6). Correlation patterns between metabolites and transcripts were similar among the organic acids with the exception of citric, benzoic and shikimic acid. A significant proportion of organic acids share similar patterns of abundance across samples; however, citric, benzoic and shikimic acid do not. Similarly, three amino acids (aspartic acid, threonine and an unidentified amino acid) had similar correlation patterns; whereas, the correlation pattern for isoleucine was distinct. Unlike the other three amino acids, isoleucine increased significantly in abundance in response to water-deficit with a more pronounced increase at the mid-day time point. These results suggest that the regulatory control of the metabolites with similar patterns of expression may be shared; whereas, the metabolites with distinct correlation patterns are likely influenced by distinct molecular mechanisms.

Among the transcripts significantly correlated with at least one metabolite, enrichment for GO terms among transcripts was determined. For transcripts with increased transcript abundance in response to drought and correlated with at least one metabolite (n = 404), four significant enriched GO biological process terms were identified: ‘proline metabolic process’ (GO:0006560), ‘arginine metabolic process’ (GO:0006525), ‘galactose metabolic process’ (GO: 0006012) and ‘serine family amino acid metabolic process’ (GO:0009069; Additional file 1: Table S6). A total of 13 significant GO terms were identified. Among transcripts that had decreased transcript abundance in response to drought and were correlated with at least one metabolite (n = 343), 15 significantly enriched GO terms were identified. For GO terms associated with biological process, ‘serine family amino acid metabolic process’ (GO:0009069), ‘tyrosine metabolic process’ (GO:0006570) and ‘aromatic amino acid family metabolic process’ (GO:0009072) were significantly enriched.

Functional annotation of the correlated transcripts and metabolites revealed pathways that were perturbed by water withdrawal (Additional file 7: Figure S6). A functional class related to starch and sucrose metabolism (pop00500) was overrepresented among the transcripts that are correlated with two identified 5C sugars and glucose (Additional file 7: Figure S6). Photosynthesis-related categories (pop00195 and pop00196) were highly associated with malic acid, raffinose and galactinol (Additional file 7: Figure S6).

In spinach, raffinose accumulation reduced electron and cyclic photophosphorylation in photosynthesis [49], and it has been hypothesised that raffinose and other RFOs play an important role in the protection of cellular metabolism, especially photosynthesis in chloroplasts in *Arabidopsis* [17]. Evidence herein suggests there may be a functional relationship in *P. balsamifera* between raffinose accumulation and transcripts associated with photosynthesis. An association between photosynthetic metabolic processes and RFO accumulation may highlight unique relationships that can be garnered from transcriptome-metabolome relationships in *Populus*.

### Energy metabolism and secondary metabolite biosynthesis varied in a genotypic-dependent manner in response to drought

Galactinol accumulation varied in response to water-deficit stress in genotype AP-1006 [log_2_ (fold-change) = −0.4526]; whereas galactinol accumulated consistently in the other genotypes. Raffinose accumulation was significant in water-deficit-treated plants, with the exception of trees of the genotype AP-2300. There was drought-responsive variation in transcript accumulation of genes hypothesised to be involved in the galactose metabolism pathway. All genotypes showed increased abundance of transcripts corresponding to galactinol synthase (EC:3.4.1.123), raffinose synthase (EC:2.4.1.82) and stachyose synthase (EC: 2.4.1.67; Additional file 8: Figure S7). Galactinol synthase transcript accumulation varied in magnitude in response to water-deficit conditions among the six genotypes, with the largest increase in transcript accumulation observed in genotypes AP-2278 and AP-1006.

Elevated levels of RFOs in *Arabidopsis* plants increased drought tolerance, highlighting the importance of these oligosaccharides in the response to osmotic-stress [20]. Increased accumulation of raffinose has been observed in desiccation tolerant seeds [50], chloroplasts of frost-hardy *Brassica oleracea* leaves [49], and in *Populus* leaves exposed to osmotic stress [16,25]. Increased transcript abundance of galactinol synthase and raffinose synthase has been observed in response to drought in *Arabidopsis* [20,51] and *Populus* [14,25]*.*

Mounting evidence suggests that the role of raffinose and other RFOs is consistent across species; however, the magnitude of change is variable, as was observed among the six *P. balsamifera* genotypes reported herein. Similarly, in four *Populus* hybrids, variable raffinose and galactinol content was shown under drought [16]. This suggests the existence of genotypic specific metabolite profiles related to these oligosaccharides, and that the level of accumulation may influence the overall drought response. Moreover, the data suggest that AP-1006 may not accumulate elevated levels of galactinol in response to drought; faster metabolism turnover or flux through this pathway may be of lower importance.

Unique relationships were also observed in the citrate cycle (TCA) pathway (KEGG, pop00020). TCA cycle intermediates. For example, succinic and malic acid, show significant variation with respect T and TxG (Additional file 1: Table S3). The metabolic rate of the TCA cycle is known to be influenced by drought [52]. The magnitude change between well watered and water-deficit treated samples for transcripts associated with the TCA cycle varied among genotypes. Citrate synthase (EC:2.3.3.1) had increased transcript accumulation in water-deficit-treated samples of AP-947, AP-1006, AP-2278 and AP-2300; however, decreased transcript accumulation was observed in the other genotypes (Additional file 9: Figure S8). Similarly, malate dehydrogenase (EC:1.1.1.37) had <1 log_2_ (fold-change) in response to drought in AP-1006 and AP-2298, whereas >1 log_2_ (fold-change) increase was observed in AP-947 and AP-2278. In *Arabidopsis*, malate dehydrogenase demonstrated increased transcript accumulation in response to drought, cold or high-salinity stress [19]; however, the variation in the genotypic response in *P. balsamifera* highlights the complexity in this response.

**Figure 5:**
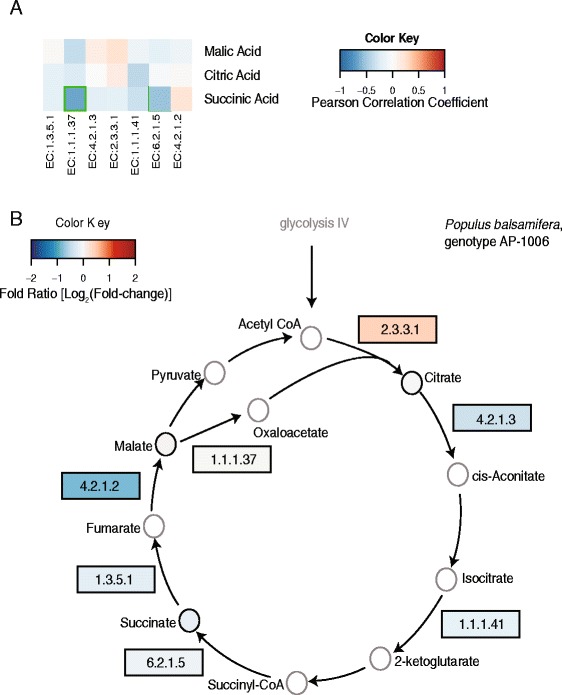
Pathway analysis related to the citric cycle (TCA cycle). **(A)** Correlation among selected transcripts and metabolites from the KEGG pathway pop00020 ‘Citrate cycle (TCA cycle)’ for genotype AP-1006. Colors represent Pearson correlation value. Red indicates positive correlation and blue represents negative correlation values. **(B)** Map displays selected steps from citrate cycle pathway. Colours indicate fold-change in transcript or metabolite abundance between water-deficit and well watered treated samples for genotype AP-1006; red indicates higher abundance in water-deficit-treated samples and blue indicates lower abundance in water-deficit-treated samples. Enzymes are given as EC numbers. EC 1.1.1.37, malate dehydrogenase; EC:1.1.1.41, isocitrate dehydrogenase (NAD+); EC:1.3.5.1, succinate dehydrogenase; EC:2.3.3.1, citrate synthase; EC:5.2.1.2, fumarate hydratase, EC: 5.2.1.3, aconitate hydratase, EC: 6.2.1.5, succinate-CoA ligase, beta subunit.

Similar to other genotypes, variations among genes involved in the TCA metabolic pathway were observed in genotype AP-1006 (Figure 5A). Pair-wise comparisons within the TCA cycle for select transcripts and metabolites found weak relationships among transcripts, and malic and citric acid accumulation profiles for AP-1006 (Figure 5B); however, succinic acid and malate dehydrogenase (EC:1.1.1.37) were significantly negatively correlated (r = −0.67, *P =* 0.0204) in genotype AP-1006 (Figure 5B). Pathway analysis highlights the influence of genotype on the drought-induced modifications to the TCA cycle in AP-1006, and, more broadly in *P. balsamifera*.

Comparative pathway analysis among genotypes has proved useful in *Populus*. In two different genotypes of *Populus* with varying salt-tolerance, pathway analysis revealed different mechanisms of tolerance between the two genotypes. Janz et al. [25] found that the salt-tolerant *Populus eupharatica* demonstrated moderate transcriptome changes in response to stress when compared to a salt-sensitive *Populus* hybrid. However, stress tolerance in *P. eupharatica* was not dependent on transcriptome modification under conditions of stress; instead, it was linked to greater energy requirements for cellular metabolism [25]. In *P. balsamifera* there are varying degrees of transcriptional remodelling in response to drought among genotypes; however, further analysis is required to understand the subtleties in these differences.

### Network analysis illuminated the nature of genotype-specific responses to drought

To identify genotype-specific transcriptome alterations, a network was created including all genes that were deemed significantly differentially expressed in a T-main effect manner for each genotype using WGCNA [31]. Weighted Pearson correlation matrices were calculated and used to determine topological overlap (TO) among genes. The TO calculated in WGCNA measured connectivity of a gene within a network relative to its neighbours. HCA based on the TO scores for all genes in the drought transcriptome grouped genes with equivalent transcript abundance profiles across all samples.

Overall, 10 network modules with equivalent transcript abundance patterns were identified. Many network modules were similar across genotypes. For example, a significant proportion of the network modules from AP-1006 were preserved in the other five genotypes (Table 4). AP-1006 was chosen as a reference because the transcriptome of AP-1006 had the highest magnitude change in response to drought relative to the other five genotypes. Not surprisingly, all of the modules were highly correlated with treatment; whereas only three were significantly correlated with time of day (M2_1006, M5_1006 and M8_1006; Table 5). Notably, M3_1006 (black) was shared between AP-1006 and AP-2278 with 62% overlap with respect to gene membership (Table 4). Among those transcripts belonging to M3_1006, there was an overrepresentation of transcripts involved in ‘intracellular signalling cascade (GO: 0007242)’. M5_1006 (brown) demonstrated a high degree of overlap among genotypes, with the exception of AP-2298. Functional characterization of M5_1006 revealed that the module was made up of genes that are often associated with drought responses in plants, and included an overrepresentation of GO terms such as: ‘response to abiotic stimulus (GO:0009628)’, ‘cellular catabolic process (GO: 0044248)’ and ‘response to water deprivation (GO: 0009414)’. The high degree of overlap between modules identified for AP-1006 and the other genotypes validated the presence of a highly conserved drought transcriptome in *P. balsamifera*.

**Table 4:** **Module membership in the drought transcriptome network of AP-1006 and preservation of drought modules among the other genotypes**

**Colour**	**Module identified in AP-1006**	**Number of module members (genes)**	**AP-947**	**AP-1005**	**AP-2278**	**AP-2298**	**AP-2300**
yellow	M1_1006	192	-	68	121	-	-
blue	M2_1006	355	49	387	242	-	-
black	M3_1006	93	-	-	58	-	-
turquoise	M4_1006	399	40	309	140	44	244
brown	M5_1006	199	48	283	183	-	38
green	M6_1006	188	-	140	149	97	51
pink	M7_1006	72	-	201	81	-	-
red	M8_1006	111	74	128	53	-	-

**Table 5:** **Module-treatment or -time of day relationships of the** ***P. balsamifera*** **(AP-1006) drought transcriptome**

**Module AP-1006**	**Treatment (T)**	**Time of day (D)**
M1_1006	−0.833*	−0.484
M2_1006	−0.662*	0.709*
M3_1006	−0.77*	0.432
M4_1006	−0.952*	0.164
M5_1006	0.759*	−0.638*
M6_1006	0.983*	−0.038
M7_1006	0.928*	0.342
M8_1006	0.699*	0.708*

Columns 2 and 3 represent the correlation between the mean expression of the module and the experimental factor (T or D). *p value < 0.05.

**Figure 6:**
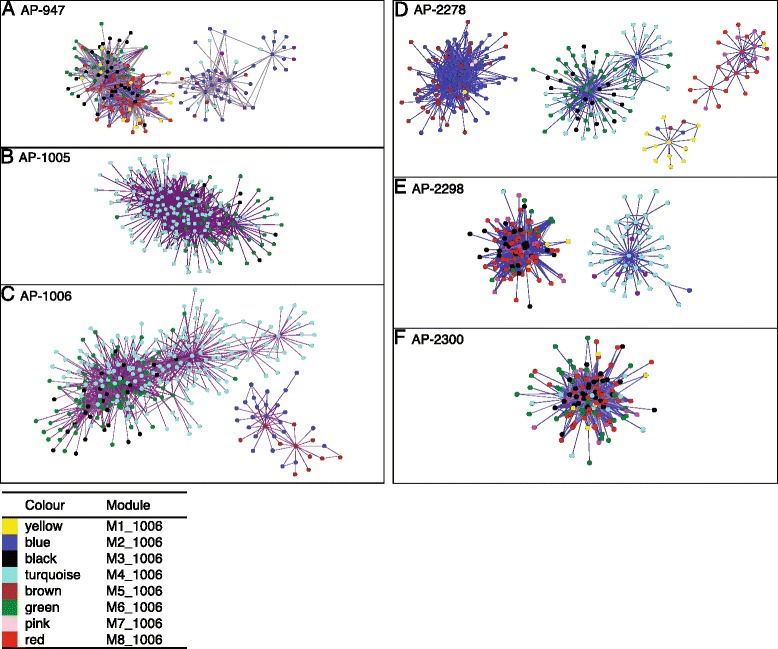
Transcript correlation networks obtained from WGCNA for **(A)** AP-947, **(B)** AP-1005, **(C)** AP-1006, **(D)** AP-2278, **(E)** AP-2298 and **(F)** AP-2300. The top 1000 interactions for each genotype are represented. Nodes in the graphs represent individual transcripts that connect via edges to other transcripts. Each node is colored according to the modules defined in Table 4.

Although there was a high degree of network module preservation among genotypes, organisation within modules varied among genotypes. When visualising the top (n = 1000) network connections of each genotype, and labelling the nodes according to their module membership within the drought transcriptome, two general observations could be made (Figure 6). First, transcript connectivity varied among genotypes. In certain genotypes, there was a higher degree of topological overlap between individual genes (nodes), as indicated by the colour of the edges. Nodes connected with a higher TO are indicated with a red/purple colour, whereas lower TO is indicated with a blue colour. For example, modules found in AP-1005 demonstrate higher TO indicating stronger connectivity among nodes and modules as compared to genotype AP-2278. Interconnectedness among genes in AP-1005 are more tightly correlated as compared to other genotypes (Figure 6). Second, the importance of any given module varied among genotypes. For example, the nodes of top network connections in genotype AP-1005 were from module M4_1006 and M6_1006, whereas genotype AP-947 had nodes that belonged to many other modules. More specifically, genes that played a more central “hub” role in the drought transcriptome networks varied among genotypes.

### AP-1006 had a genotype-specific transcriptome response to drought

Due to the uniqueness of the transcriptome of AP-1006, genes central to the network modules in this genotype were interrogated. The largest magnitude of change was observed in the drought transcriptome for AP-1006. Transcript abundance of hub genes in AP-1006 revealed samples clustered according to treatment; however, transcript abundance profiles were more similar between well watered samples, regardless of time of day. The absolute magnitude change in abundance of transcripts central to the network modules in AP-1006 was significantly higher than the magnitude change of the transcripts in the other genotypes [absolute log_2_ (fold-change) AP-1006 = 2.36]. Many hub transcripts had significant changes in transcript abundance in response to drought in AP-1006. There are 195 hub transcripts (TO Network Ratio > 0.5) that have decreased abundance in response to drought in AP-1006; whereas there are 104 hub transcripts that had increased abundance in response to drought.

Enrichment of GO terms within the set of central network hub transcripts from genotype AP-1006 revealed the components of the genotype-specific drought transcriptome. For example, transcripts implicated in the response to stress and stimuli were enriched. Of the hub transcripts with significant declines in abundance, genes implicated in carbohydrate metabolism were enriched, including those with GO terms for: sucrose (GO:0005985), starch (GO: 0005982) and disaccharide (GO: 0005984) metabolic processes (Additional file 10: Figure S9A). Conversely, core hub transcripts with increased accumulation in response to drought in AP1006 were enriched for biological processes, including response to stimulus (GO:0050896) and stress (GO: 0006950) as well as transport (GO:0006910) and regulation of cellular processes (GO:0050794; Additional file 10: Figure S9B). However, it should be noted that there was a large proportion of transcripts that had unknown function. The transcripts that played a central role in the network organisation of the drought transcriptome in AP-1006 were likely important regulators of the drought response, and the analysis of transcript co-expression relationships may help with functional annotation; albeit, not with immediate interpretation.

### There were strong correlates between specific transcript-metabolite pairs in response to drought in AP-1006

Strong correlations between transcript and metabolite abundance in response to drought in AP-1006 were observed in metabolic pathways, including: ‘plant hormone signal transduction’, ‘arginine and proline metabolism’, and ‘glycolysis/gluconeogenesis’ (Additional file 11: Figure S10). As previously noted, one of the largest magnitude change in transcript abundance was observed in AP-1006 (Figure S5). Transcripts, including those encoding genes homologues to *Arabidopsis thaliana* RAC-like 2 protein (ARAC2) and IRREGULAR XYLEM 9 (IRX9) had significantly larger fold-change decrease in transcript abundance in response to water-deficit conditions as compared to other genotypes. Conversely, several transcripts annotated as universal stress proteins, or those involved in hormone signalling had significantly higher transcript abundance in AP-1006 in response to water-deficit stress. Correlation network analysis revealed core transcripts that might have played a role in the underlying mechanisms regulating metabolite accumulation in AP-1006. Transcripts most strongly correlated with metabolite levels were identified. Although no particular class of metabolites or transcripts appeared specific to AP-1006, a large number of transcripts highly correlated with succinic acid, raffinose and galactinol accumulation (Additional file 1: Table S7). For example, strong positive correlations were observed between raffinose, galactinol and a photosystem II reaction center PsbP family protein (r = 0.871 and 0.835, respectively; Additional file 1: Table S7). Strong correlations between drought responsive metabolites and transcripts reveal pathways that may be of importance in the drought tolerance mechanisms in a genotype.

## Conclusion

The metabolomics response to drought in *Populus balsamifera* in these experiments was complex, and the variation within the metabolome was highlighted by variation among genotypes and between time-of-day responses. Although common drought-responsive metabolites could be identified across all six *P. balsamifera* genotypes, a significant proportion of metabolites varied in a genotype or time-of-day dependent manner. The complexity of the genotype-metabolite relationship was notable, and likely attributable to the function of many genes, the environment and their interaction. Integrating transcriptome and metabolome data identified significant metabolite-gene correlation, whereby biologically meaningful correlations were derived. Metabolite-transcript relationships from the same and different pathways were identified, and may be useful for future elucidation of important drought response mechanisms. Integration of the transcriptome and metabolome data at individual pathway levels revealed variation in metabolite flux and transcript accumulation among genotypes in energy and galactose metabolism.

The impacts of environmental stress on forest health and productivity are becoming of increasing concern. The results presented herein demonstrate that future experiments aimed at understanding the complexities of the responses of forest trees to environmental stimuli must take into consideration the intraspecific variation in these responses. Although common drought responses among genotypes of *P. balsamifera* could be identified, significant intraspecific variation was observed. The intraspecific variation in the molecular strategies that underpin the responses to drought among genotypes may have an important role in the maintenance of forest health and productivity, particularly amidst future challenges imposed by reduced forest integrity and fluctuating environmental conditions.

## Availability of supporting data

The microarray data set supporting the results of this article is available in the Gene Expression Omnibus (GEO) database of the National Center for Biotechnology Information of the USA as series GSE21171 (http://www.ncbi.nlm.nih.gov/geo/query/acc.cgi?acc=GSE21171). The metabolite data set supporting the results of this article is included within the article, and can be found in the Supplemental Information (Additional file 1: Table S1).

## Additional files

Additional file 1: Table S1. Original data matrix for all metabolites (n = 87). **Table S2.** Summary statistics for all metabolites (n = 87). Mean peak intensity values represented for all samples, wet samples and dry samples ± standard deviation. Relative abundance between water-deficit and well watered samples represented as log_2_ (fold-ratio); positive values indicate increased abundance in water-deficit conditions, whereas negative values indicate decreased abundance in water-deficit conditions relative to control conditions. *p* values for treatment (T) main effect as calculated per the factorial ANOVA; metabolites significant for treatment main effect denoted with an * (FDR < 0.05). Metabolite classes are as follows: AA = Amino Acid; C = Carbohydrate; P = Phenolic, SA = Sugar Alcohol, and NI = Not Identified. **Table S3.** ANOVA results: metabolite abundance. Significance: ****P* < 0.001; ***P* < 0.01; **P* < 0.05; . *P* < 0.1 **Table S4.** Pair-wise comparisons among genotypes for absolute magnitude log_2_ (fold-change) variation for the drouglht metabolome (n = 40). Bolded asterisks indicate significant differences according to Bonferroni’s test (*P* < 0.05). **Table S5.** Pair-wise comparisons among genotypes for absolute magnitude log_2_ (fold-change) variation for the drought transcriptome (n = 763). Bolded asterisks indicate significant differences according to Bonferroni’s test (*P* < 0.05). **Table S6.** GO term enrichment among transcripts that are significantly correlated with at least one metabolite. (A) For transcripts with increased abundance in water-deficit treated samples and (B) for transcripts with decreased abundance in water-deficit treated samples. **Table S7.** Pair-wise M:T Spearman correlation values for genotype AP-1006. Additional file 2: Figure S1. Dendrogram obtained after HCA of the metabolic profiles of six *P. balsamifera* genotypes. Metabolite profiles collected for six genotypes under well watered and water-deficit conditions at mid-day and pre-dawn time point. Rows represent specific metabolites (n = 87). Columns represent mean intensity of all replicates for each genotype, treatment and time of day sample. Plotted values are the mean of n = 4–10 replicates for each sample. Metabolite classes: AA = Amino Acid; C = Carbohydrate; P = Phenolic, SA = Sugar Alcohol. NI = Not Identified. Additional file 3: Figure S2. HCA reveals 13 significant clusters (P < 0.05). Significant clusters are numbered (I through XIII) for identification. Hierarchical clustering was done using pvclust (Suzuki and Shimodaira [29]), with a correlation distance measure and a complete agglomerative clustering method. Additional file 4: Figure S3. Metabolites that are significant for a treatment (T):time-of-day (D) interaction (*P* <0.05, n = 15). Additional file 5: Figure S4. Metabolites that are significant for a treatment (T):genotype (G) interaction (*P* <0.05, n = 41). Additional file 6: Figure S5. Box-plot illustrating the interplay of genotype and treatment in shaping the drought metabolome and transcriptome of six *P. balsamifera* genotypes. The average absolute log_2_ (fold-change) between well watered and water-deficit-treated samples for all (A) metabolites (n = 40; *P* < 0.05) and (B) transcripts (n = 1848; *p* < 0.05) with significant variation in their abundance between treatment conditions at the mid-day (MD) time point. Additional file 7: Figure S6. Heatmap of drought responsive transcript and metabolite correlations. Of all the drought responsive transcripts, 747 unique transcripts were correlated with at least one metabolite (|*r*| > 0.6; *p* < 0.05). The rows in the heatmap represent metabolites, and the columns represent transcripts. The columns are clustered based on their expression across samples, and the metabolites are grouped according to functional categories. Pearson correlation coefficient (*r*) are represented for each pair-wise metabolite-transcript comparison, and were calculated using R. Additional file 8: Figure S7. Pathway analysis related to the galactose metabolism. (A) Pathway map displays selected steps from galactose metabolism pathway. Colours indicate fold-change in transcript or metabolite abundance between water-deficit and well watered treated samples for all six genotypes; red indicates higher abundance in water-deficit-treated samples and blue indicates lower abundance in water-deficit-treated samples. Enzymes are given as EC numbers. EC 2.4.1.123, galactinol synthase; EC:2.4.1.82, raffinose synthase; EC:2.4.1.67, stachyose synthase. (B) Heatmap representing Spearman correlation values among transcripts related to galactose metabolism and raffinose or galactinol. Additional file 9: Figure S8. Analysis of the citrate cycle (TCA; pop00020) pathway in genotype AP-947, AP-1005, AP-2278, AP-2298 and AP-2300. (a) Correlation among select transcripts and metabolites from the KEGG pathway pop00020 ‘Citrate cycle (TCA cycle)’ for genotype AP-1006. Colors represent Pearson correlation value. Red indicates positive correlation and blue represents negative correlation values. (b) Map displays selected steps from citrate cycle pathway. Colours indicate fold-change in transcript or metabolite abundance between water-deficit and well watered treated samples; red indicates higher abundance in water-deficit treated samples and blue indicates lower abundance in water-deficit treated samples. Enzymes are given as EC numbers. EC 1.1.1.37, malate dehydrogenase; EC:1.1.1.41, isocitrate dehydrogenase (NAD+); EC:1.3.5.1, succinate dehydrogenase; EC:2.3.3.1, citrate synthase; EC:5.2.1.2, fumarate hydratase, EC: 5.2.1.3, aconitate hydratase, EC: 6.2.1.5, succinate-CoA ligase, beta subunit. Pearson correlation and pathway maps for AP-1006 can be found in Figure 5. Additional file 10: Figure S9. Overrepresentation of GO terms associated with transcripts that have (A) decreased transcript abundance in AP-1006 and (B) increased transcript abundance in AP-1006. Figures generated using AgriGO (http://bioinfo.cau.edu.cn/agriGO). Significant overrepresentation is represented by darker shaded boxes (*p* < 0.05). Additional file 11: Figure S10. Heatmap of representative functional classes (transcripts) from the correlation data. The averaged Spearman correlation value is represented for significant functional class: metabolite comparisons (coloured squares). Red indicates positive correlation, whereas blue indicates negative correlation. The number of transcripts represented in each functional group is represented in brackets.

## Abbreviations

ARAC2: Arabidopsis thaliana RAC-like 2 protein
BCAA: Branched chain amino acids
D: Time-of-day
DW: Dry weight
FDR: False discovery rate
FW: Fresh weight
G: Genotype
GC/MS: Gas chromatography/mass-spectrometry
GO: Gene ontology
HCA: Hierarchal cluster analysis
IRX9: IRREGULAR XYLEM 9
MD: Mid day
MSTFA: N-methyl-N-trimethylsilytriflouroacetamide
PD: Pre dawn
Pro: Proline
RFO: Raffinose family oligosaccharide
ROS: Reactive oxygen species
RWC: Relative water content
T: Treatment
TCA: Citrate cycle
TO: Topological overlap
Val: Valine
WGCNA: Weighted co-expression network analysis
