# Supplementary figures and images for: Poplar trees reconfigure the transcriptome and metabolome in response to drought in a genotype- and time-of-day-dependent manner
Source: BMC Genomics. 2015 Apr 21;16(1):329. doi: 10.1186/s12864-015-1535-z (PMC4437445; doi:10.1186/s12864-015-1535-z)

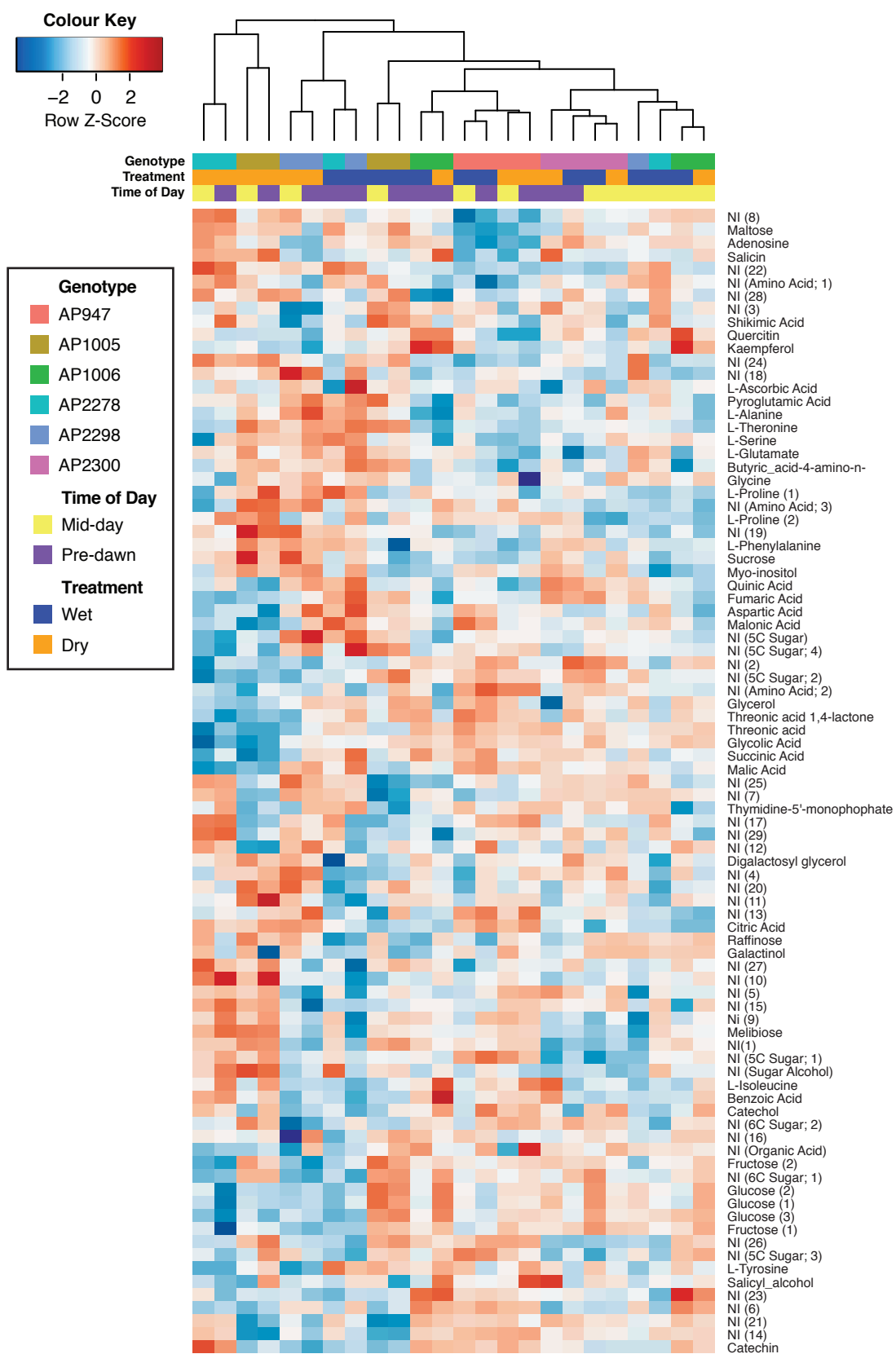

Supplementary Figure S1

Supplement: Additional file 2: Figure S1. — Dendrogram obtained after HCA of the metabolic profiles of six P. balsamifera genotypes. Metabolite profiles collected for six genotypes under well watered and water-deficit conditions at mid-day and pre-dawn time point. Rows represent specific metabolites (n = 87). Columns represent mean intensity of all replicates for each genotype, treatment and time of day sample. Plotted values are the mean of n = 4–10 replicates for each sample. Metabolite classes: AA = Amino Acid; C = Carbohydrate; P = Phenolic, SA = Sugar Alcohol. NI = Not Identified. [file 12864_2015_1535_MOESM2_ESM.pdf]

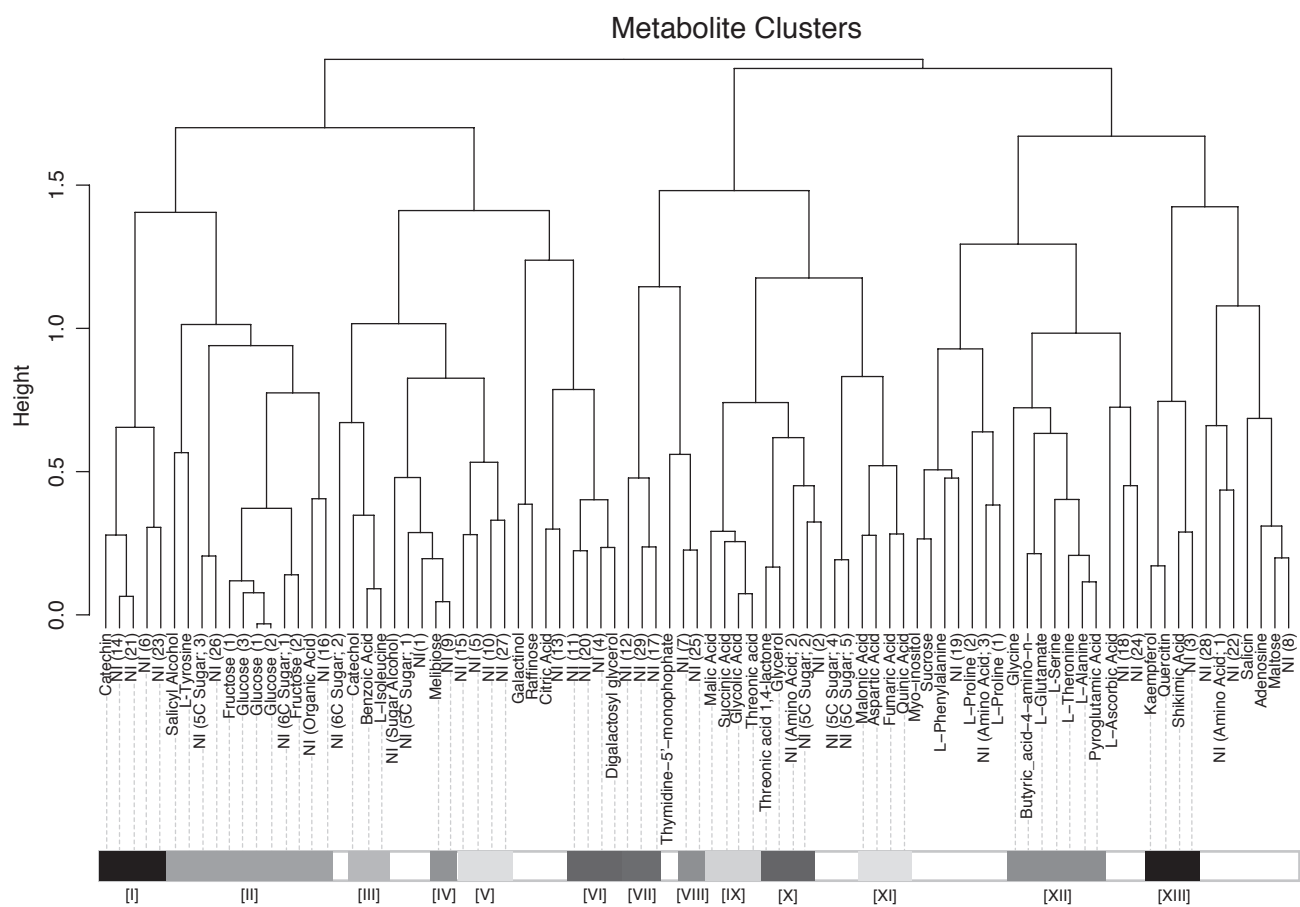

Supplementary Figure S2

Supplement: Additional file 3: Figure S2. — HCA reveals 13 significant clusters (P < 0.05). Significant clusters are numbered (I through XIII) for identification. Hierarchical clustering was done using pvclust (Suzuki and Shimodaira [29]), with a correlation distance measure and a complete agglomerative clustering method. [file 12864_2015_1535_MOESM3_ESM.pdf]

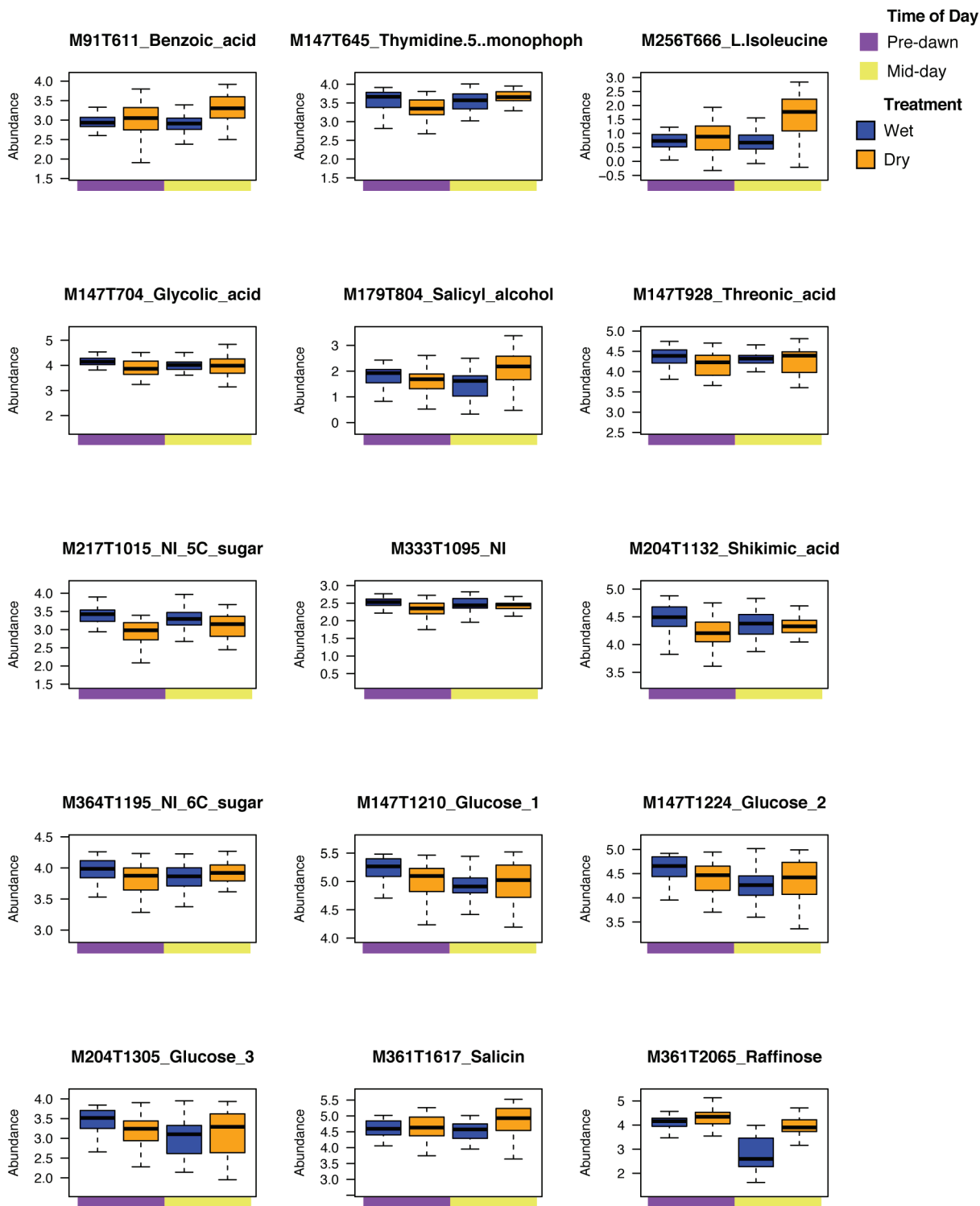

Supplementary Figure S3

Supplement: Additional file 4: Figure S3. — Metabolites that are significant for a treatment (T):time-of-day (D) interaction (P <0.05, n = 15). [file 12864_2015_1535_MOESM4_ESM.pdf]

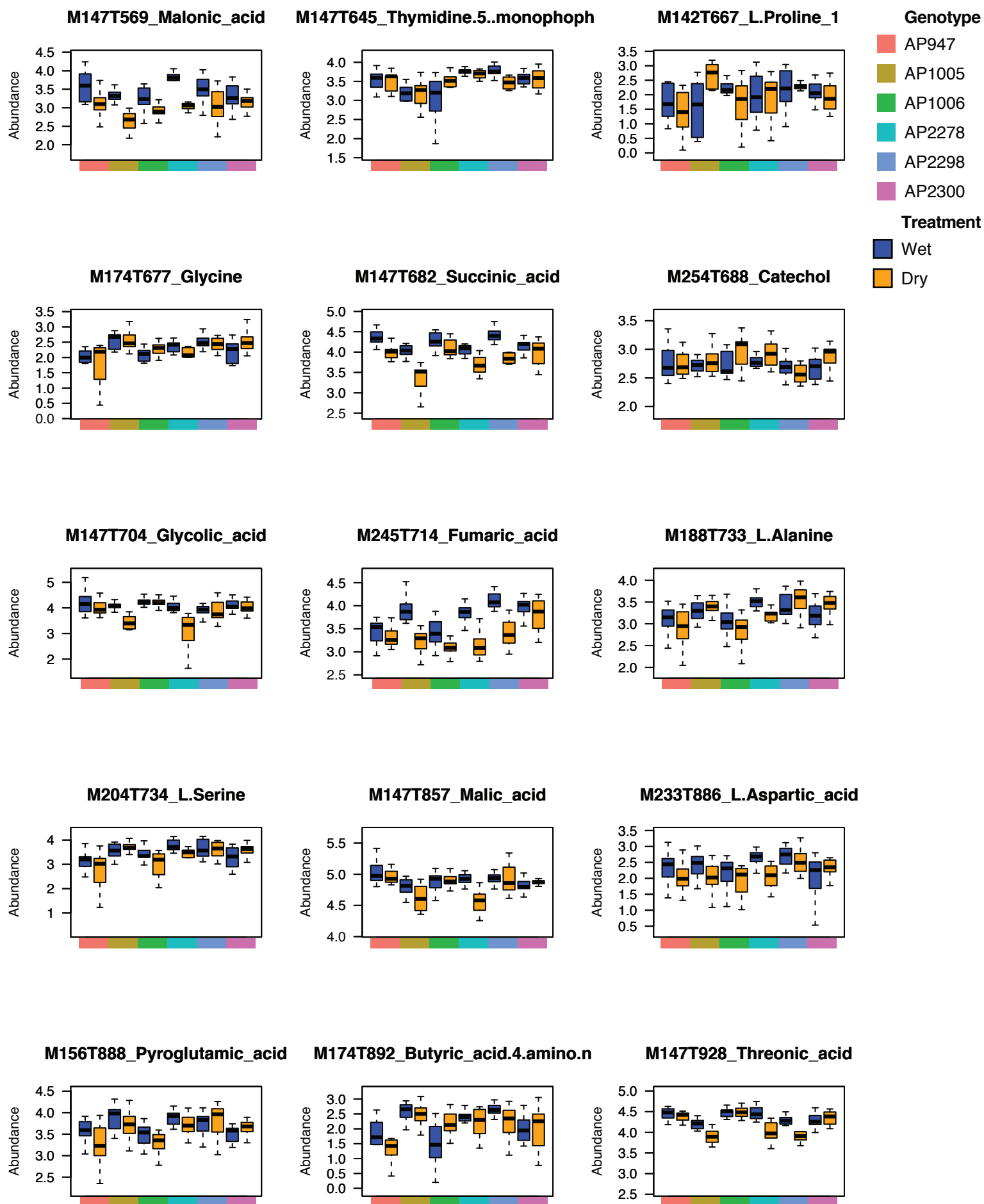

Supplementary Figure S4

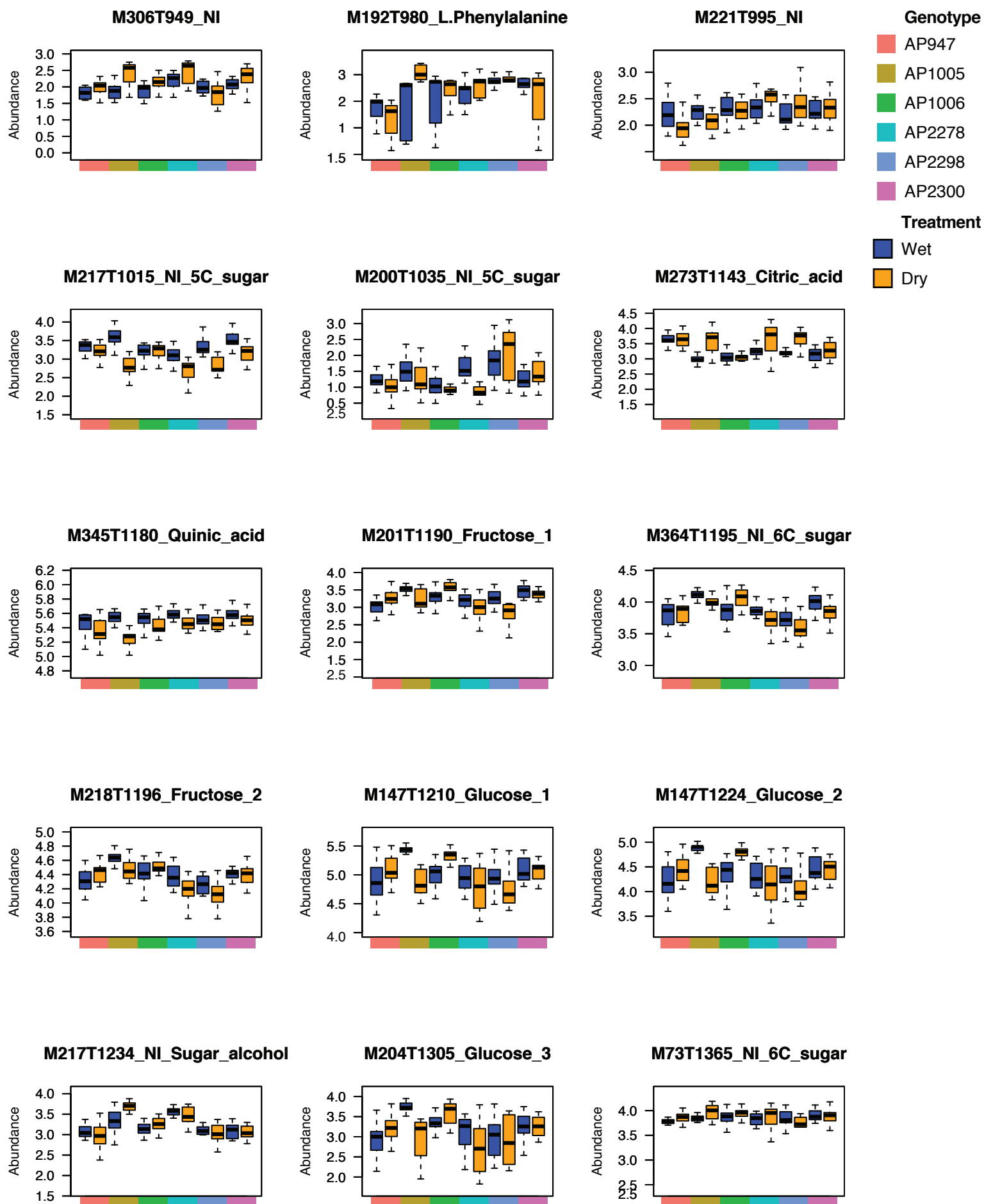

Supplementary Figure S4

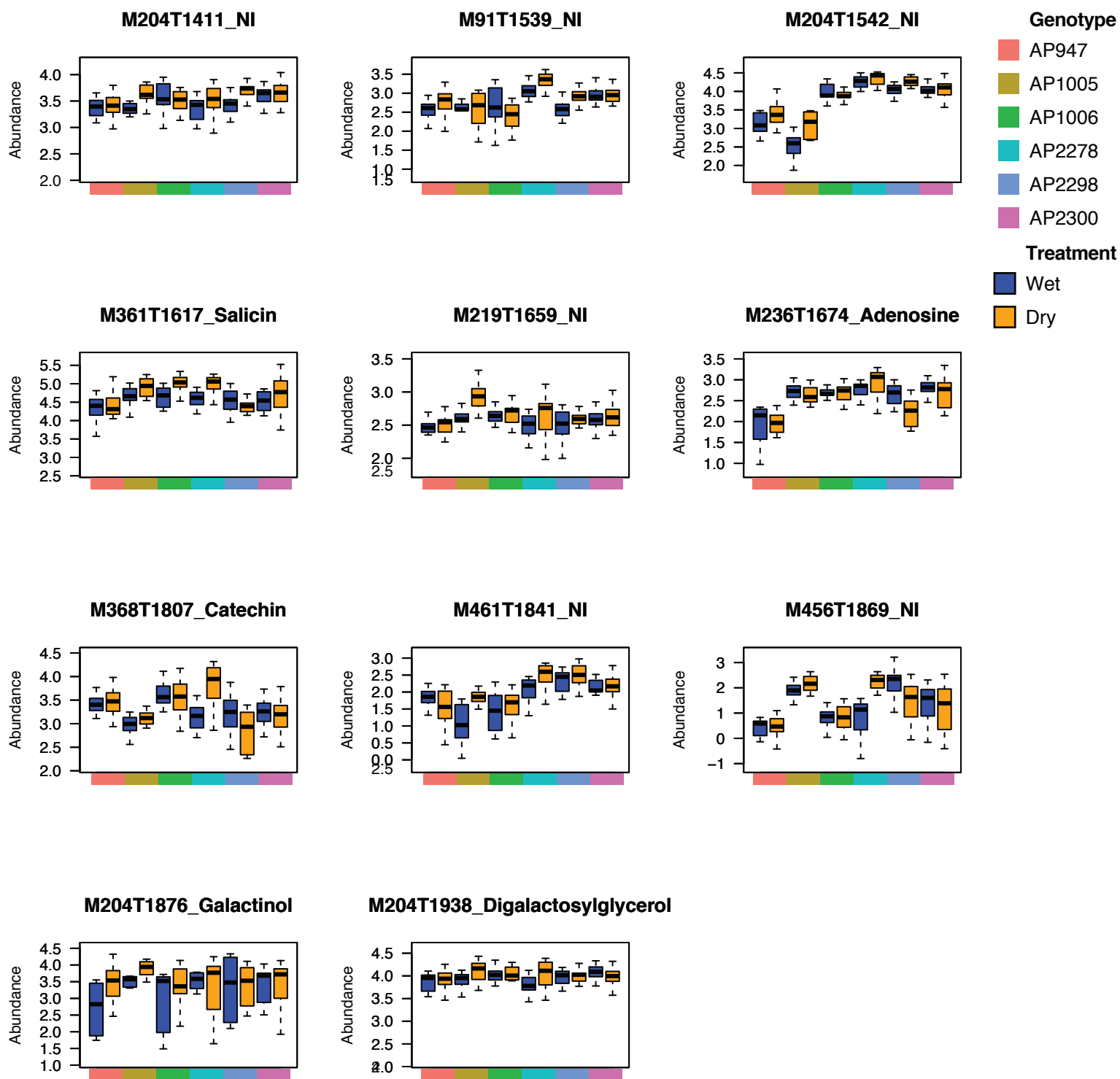

Supplementary Figure S4

Supplement: Additional file 5: Figure S4. — Metabolites that are significant for a treatment (T):genotype (G) interaction (P <0.05, n = 41). [file 12864_2015_1535_MOESM5_ESM.pdf]

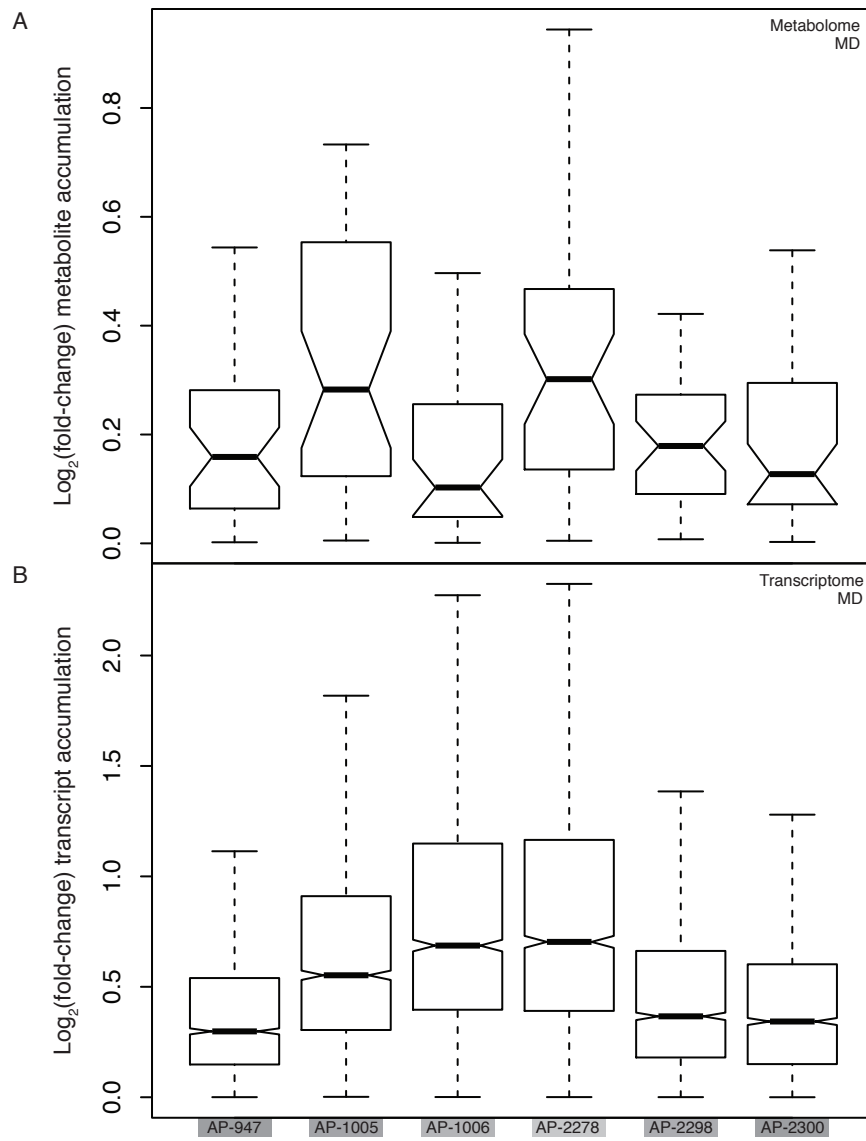

Supplementary Figure S5

Supplement: Additional file 6: Figure S5. — Box-plot illustrating the interplay of genotype and treatment in shaping the drought metabolome and transcriptome of six P. balsamifera genotypes. The average absolute log2 (fold-change) between well watered and water-deficit-treated samples for all (A) metabolites (n = 40; P < 0.05) and (B) transcripts (n = 1848; p < 0.05) with significant variation in their abundance between treatment conditions at the mid-day (MD) time point. [file 12864_2015_1535_MOESM6_ESM.pdf]

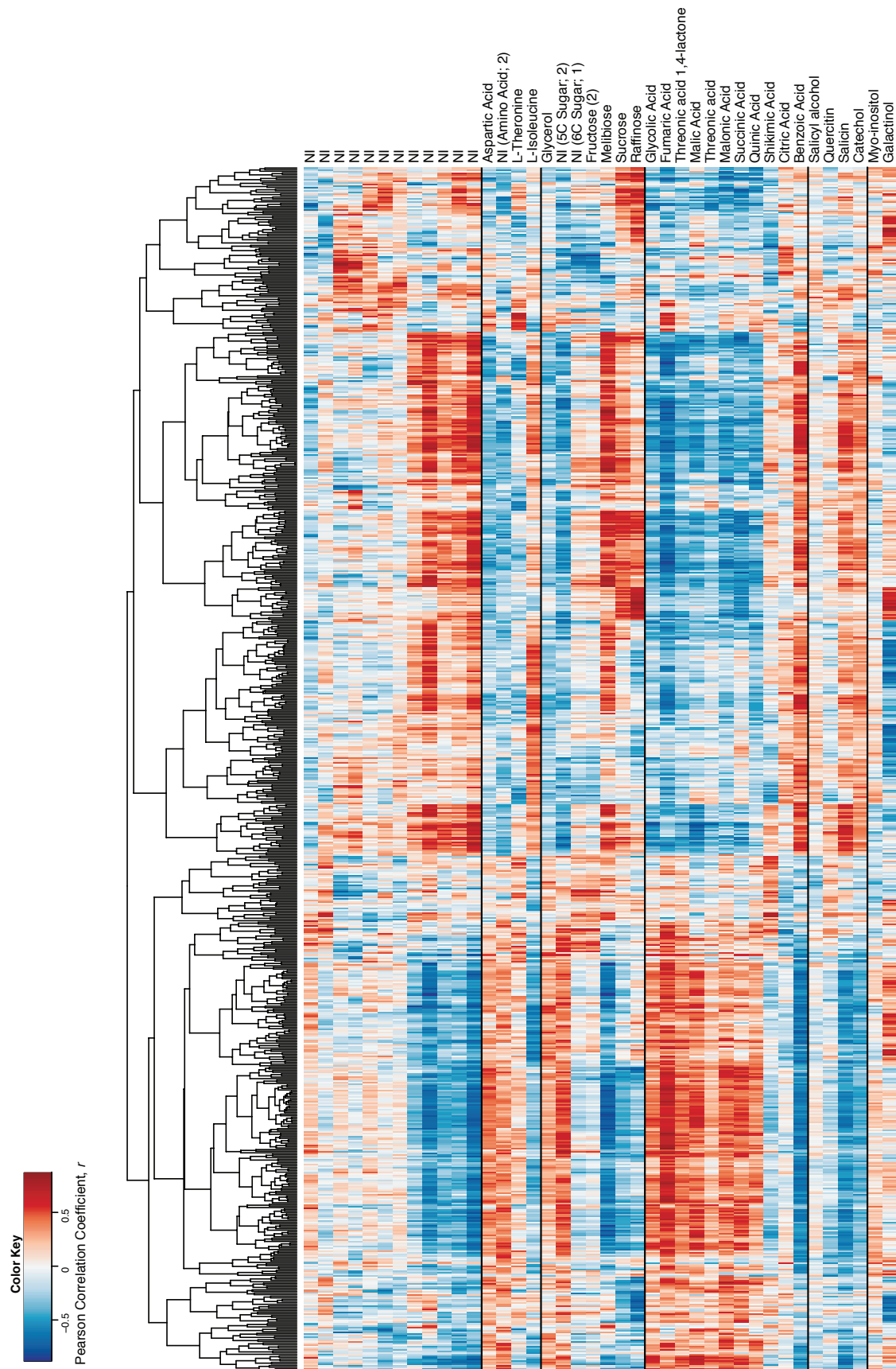

Supplementary Figure S6

Supplement: Additional file 7: Figure S6. — Heatmap of drought responsive transcript and metabolite correlations. Of all the drought responsive transcripts, 747 unique transcripts were correlated with at least one metabolite (|r| > 0.6; p < 0.05). The rows in the heatmap represent metabolites, and the columns represent transcripts. The columns are clustered based on their expression across samples, and the metabolites are grouped according to functional categories. Pearson correlation coefficient (r) are represented for each pair-wise metabolite-transcript comparison, and were calculated using R. [file 12864_2015_1535_MOESM7_ESM.pdf]

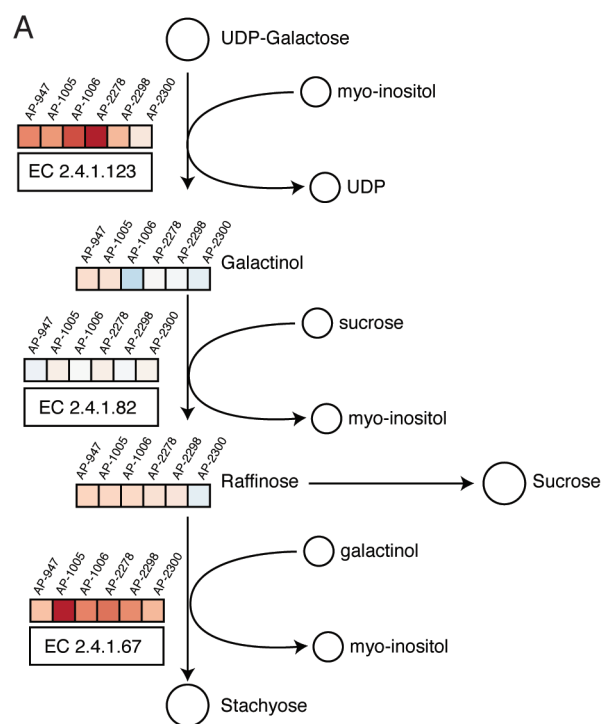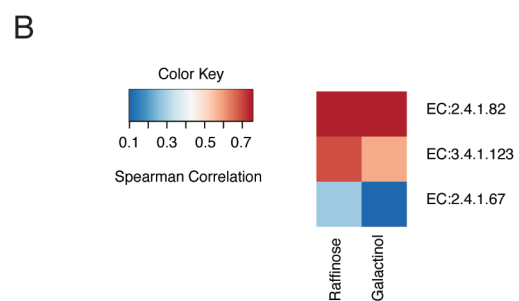

Supplementary Figure S7

Supplement: Additional file 8: Figure S7. — Pathway analysis related to the galactose metabolism. (A) Pathway map displays selected steps from galactose metabolism pathway. Colours indicate fold-change in transcript or metabolite abundance between water-deficit and well watered treated samples for all six genotypes; red indicates higher abundance in water-deficit-treated samples and blue indicates lower abundance in water-deficit-treated samples. Enzymes are given as EC numbers. EC 2.4.1.123, galactinol synthase; EC:2.4.1.82, raffinose synthase; EC:2.4.1.67, stachyose synthase. (B) Heatmap representing Spearman correlation values among transcripts related to galactose metabolism and raffinose or galactinol. [file 12864_2015_1535_MOESM8_ESM.pdf]

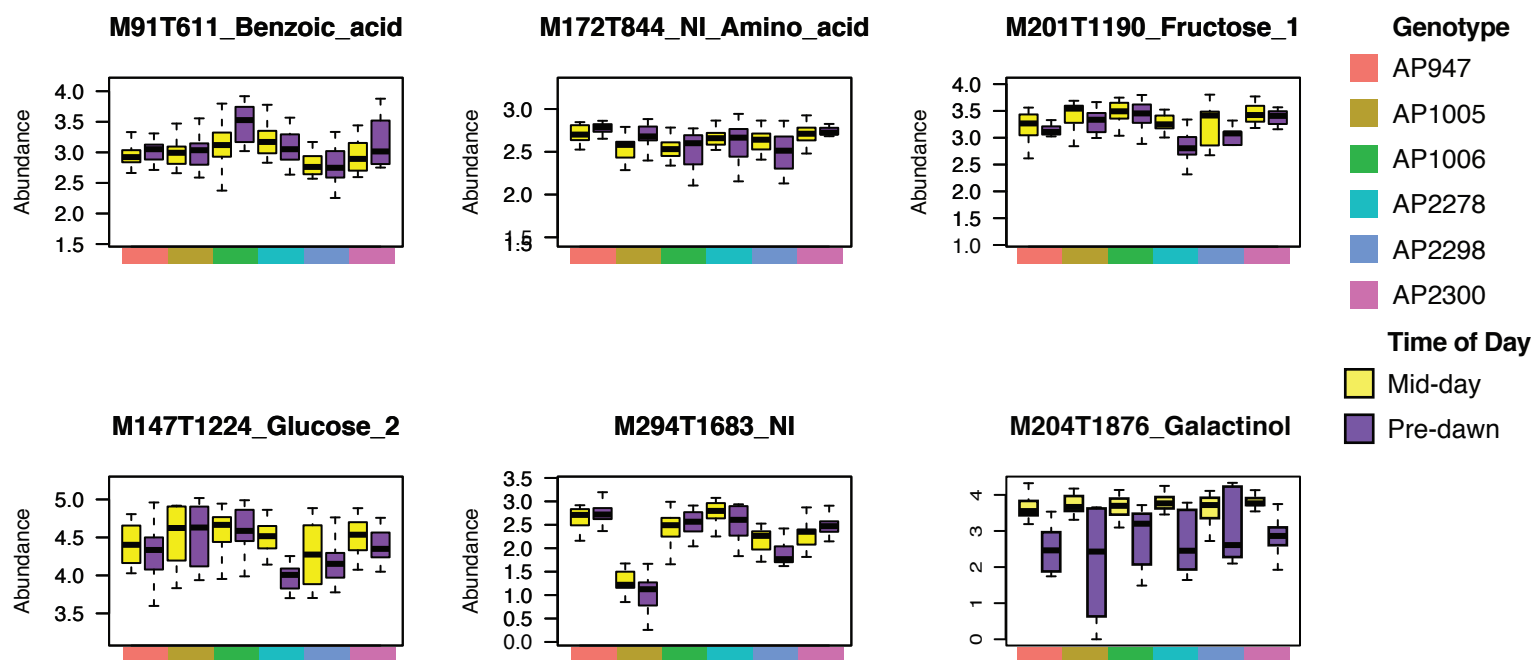

Supplementary Figure S8

Supplement: Additional file 9: Figure S8. — Analysis of the citrate cycle (TCA; pop00020) pathway in genotype AP-947, AP-1005, AP-2278, AP-2298 and AP-2300. (a) Correlation among select transcripts and metabolites from the KEGG pathway pop00020 ‘Citrate cycle (TCA cycle)’ for genotype AP-1006. Colors represent Pearson correlation value. Red indicates positive correlation and blue represents negative correlation values. (b) Map displays selected steps from citrate cycle pathway. Colours indicate fold-change in transcript or metabolite abundance between water-deficit and well watered treated samples; red indicates higher abundance in water-deficit treated samples and blue indicates lower abundance in water-deficit treated samples. Enzymes are given as EC numbers. EC 1.1.1.37, malate dehydrogenase; EC:1.1.1.41, isocitrate dehydrogenase (NAD+); EC:1.3.5.1, succinate dehydrogenase; EC:2.3.3.1, citrate synthase; EC:5.2.1.2, fumarate hydratase, EC: 5.2.1.3, aconitate hydratase, EC: 6.2.1.5, succinate-CoA ligase, beta subunit. Pearson correlation and pathway maps for AP-1006 can be found in Figure 5. [file 12864_2015_1535_MOESM9_ESM.pdf]

A

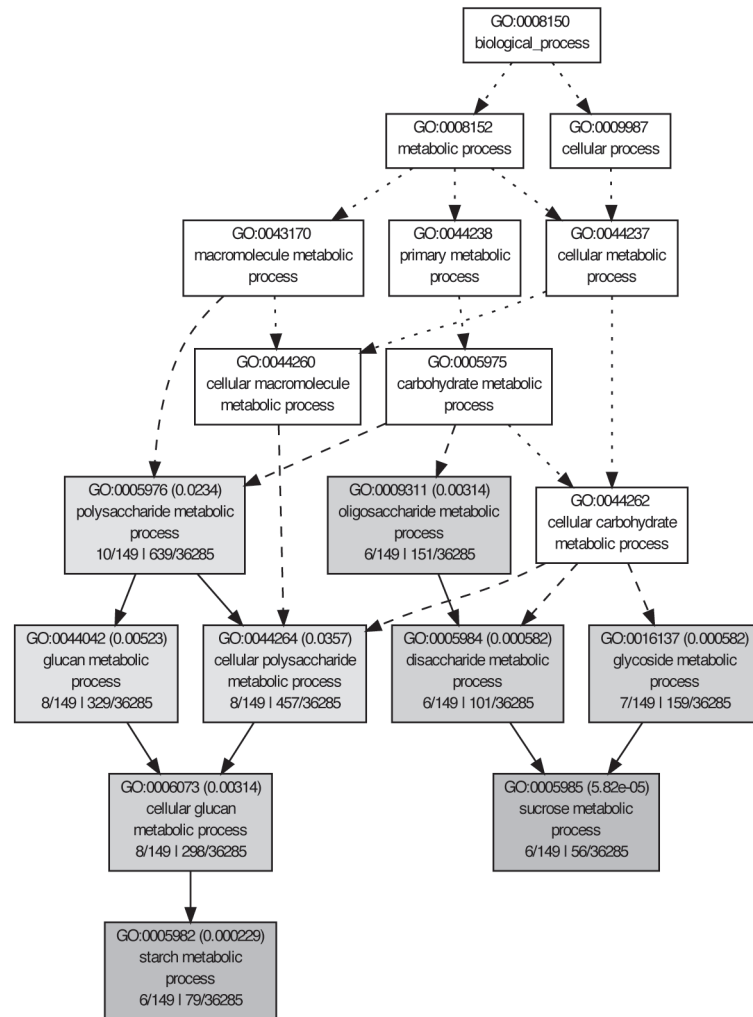

Supplementary Figure S9

B

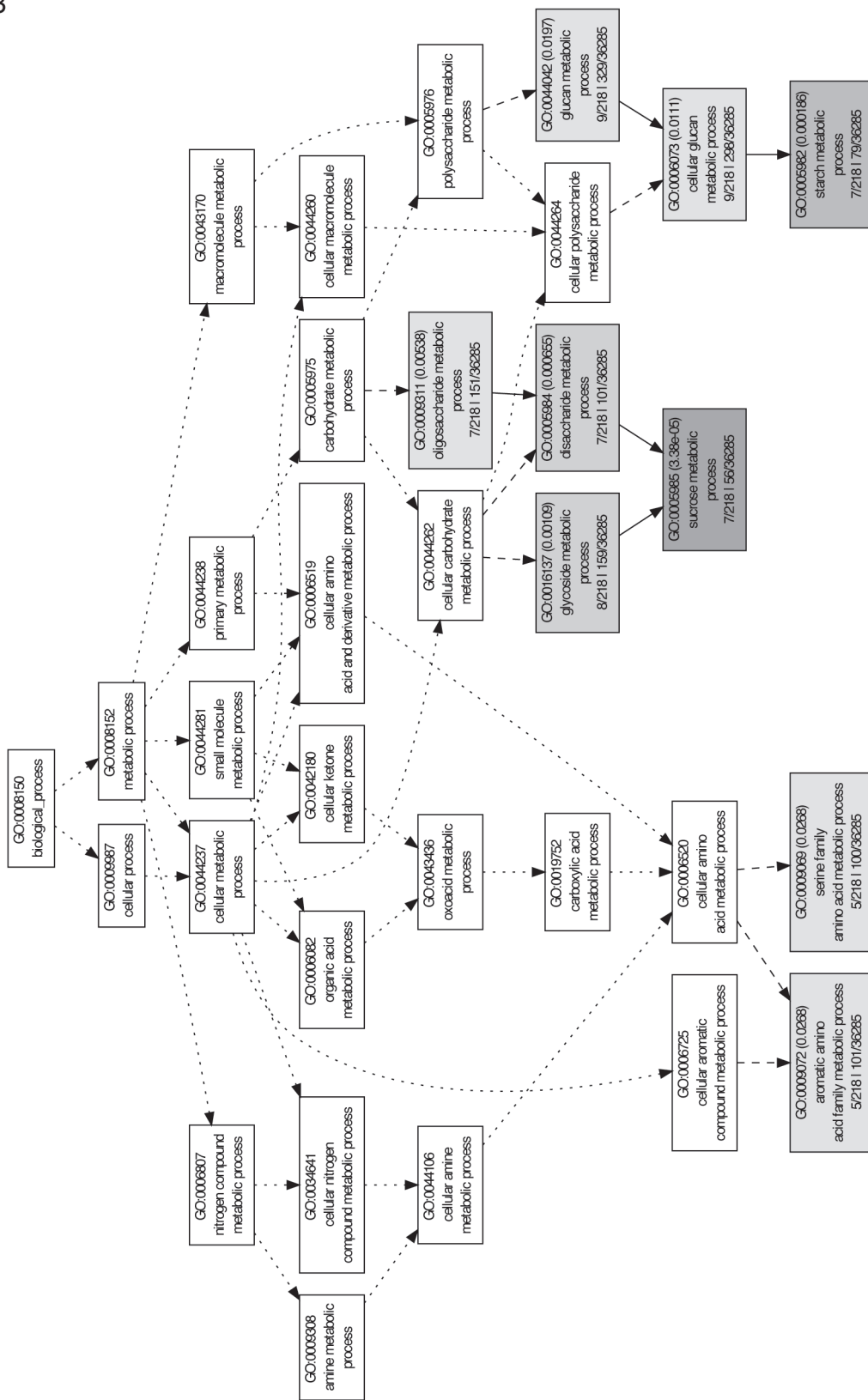

Supplementary Figure S9

Supplement: Additional file 10: Figure S9. — Overrepresentation of GO terms associated with transcripts that have (A) decreased transcript abundance in AP-1006 and (B) increased transcript abundance in AP-1006. Figures generated using AgriGO (http://bioinfo.cau.edu.cn/agriGO). Significant overrepresentation is represented by darker shaded boxes (p < 0.05). [file 12864_2015_1535_MOESM10_ESM.pdf]

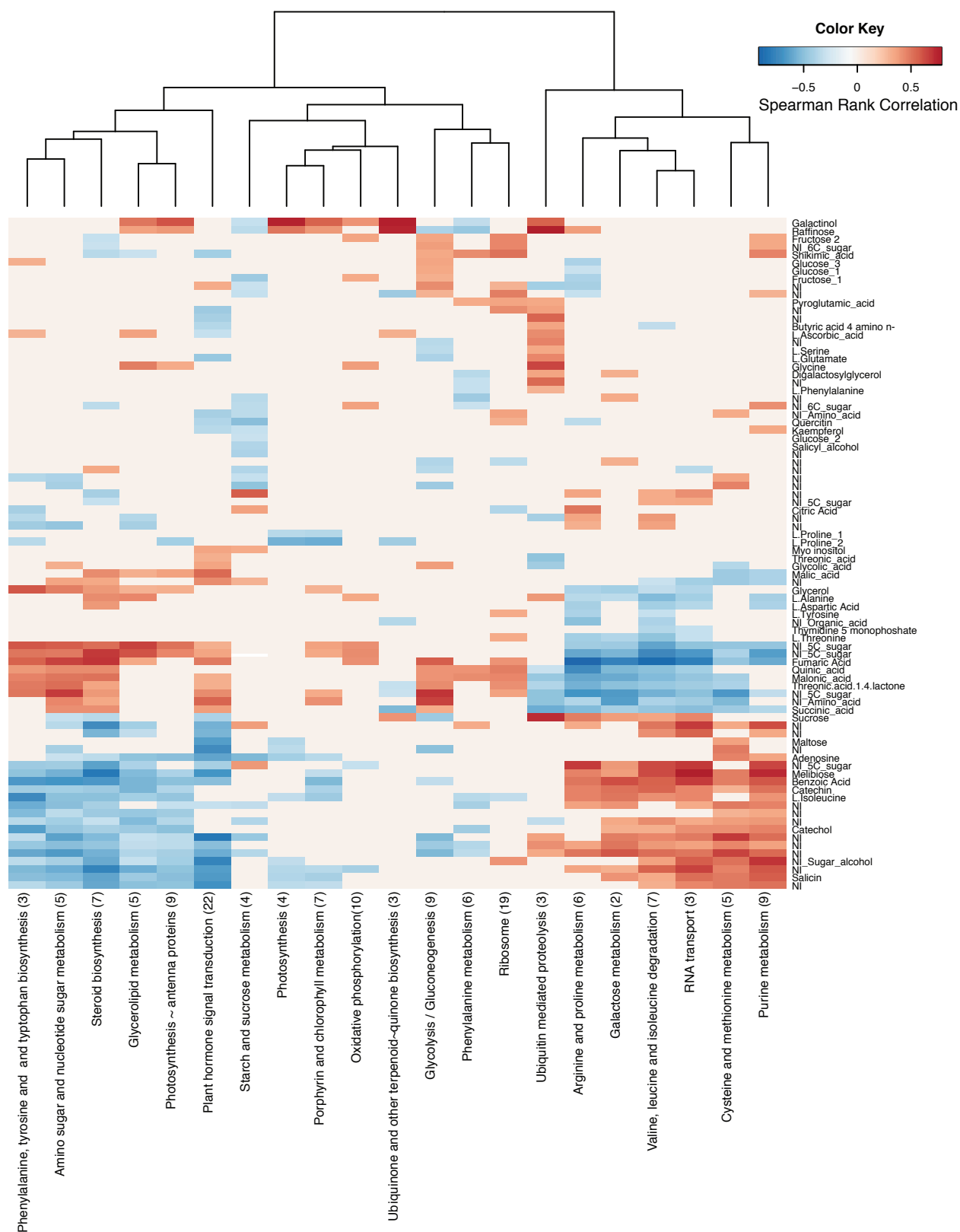

Supplementary Figure S10

Supplement: Additional file 11: Figure S10. — Heatmap of representative functional classes (transcripts) from the correlation data. The averaged Spearman correlation value is represented for significant functional class: metabolite comparisons (coloured squares). Red indicates positive correlation, whereas blue indicates negative correlation. The number of transcripts represented in each functional group is represented in brackets. [file 12864_2015_1535_MOESM11_ESM.pdf]
